# Improved Orthogonality in Naphthalimide/Cyanine Dyad Boosts Superoxide Generation: a Tumor‐Targeted Type‐I Photosensitizer for Photodynamic Therapy of Tumor by Inducing Ferroptosis

**DOI:** 10.1002/advs.202417179

**Published:** 2025-03-06

**Authors:** Guangxiao Yao, Junfeng Miao, Yingying Huo, Wei Guo

**Affiliations:** ^1^ School of Chemistry and Chemical Engineering Shanxi University Taiyuan 030006 China

**Keywords:** heptamethine cyanine, naphthalimide, photodynamic therapy, superoxide, tumors

## Abstract

It is highly desired to achieve Type‐I photosensitizer (PS) to overcome the hypoxic limitation found in most clinically used PSs. Herein, a new heavy‐atom‐free Type‐I PS **T‐BNCy5** is presented by incorporating a biotin‐modified naphthalimide (NI) unit into the *meso*‐position of a *N*‐benzyl‐functionalized, strongly photon‐capturing pentamethine cyanine (Cy5) dye. Such molecular engineering induces a rigid orthogonal geometry between NI and Cy5 units by introducing an intramolecular sandwich‐like π–π stacking assembly, which effectively promotes intersystem crossing (ISC) and greatly extends the triplet‐state lifetime (τ = 389 µs), thereby markedly improving the superoxide (O_2_^•−^)‐generating ability. In vitro assays reveal that **T‐BNCy5** specifically accumulates in mitochondria, where it not only generates O_2_^•−^ under photoirradiation but also induces the burst of the most cytotoxic hydroxy radical (HO^•^) by a cascade of biochemical reactions, ultimately triggering cell ferroptosis with the IC_50_ value up to ≈0.45 µm whether under normoxia or hypoxia. In vivo assays manifest that, benefiting from its biotin unit, **T‐BNCy5** displays a strong tumor‐targeting ability, and after a single PDT treatment, it can not only ablate the tumor almost completely but also be cleared from the body through biosafe urinary excretion, indicating its potential for future clinical translation.

## Graphical abstract

A tumor‐targeted, renal‐metabolized, and less O_2_‐dependent Type‐I photosensitizer is presented for photodynamic therapy of tumors. The formation of an intramolecular sandwich‐like π–π assembly ensures its rigid orthogonal geometry, thereby improving the superoxide (O_2_^•−^)‐generating ability, promoting the intracellular formation of hydroxy radical (HO^•^), and ultimately triggering cancer cell ferroptosis.

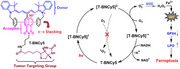

## 1 Introduction

Photodynamic therapy (PDT), as a valuable treatment modality for cancers, has garnered significant attention due to its high spatiotemporal precision and minimal invasiveness. Photosensitizer (PS), light, and molecular oxygen (O_2_) are three necessary components during PDT, which collaboratively induce phototoxicity by producing cytotoxic reactive oxygen species (ROS).^[^ ^1^ ^]^ Upon photoirradiation, PS is activated to a singlet excited state (S_1_) from the ground state (S_0_), which then decays to a triplet excited state (T_1_) via intersystem crossing (ISC). T_1_ state is a long‐lived state and could directly transfer excitation energy to surrounding O_2_ to produce singlet oxygen (^1^O_2_) (Type‐II) or drive a cascade of electron transfer to generate radical intermediates (Type‐I), such as superoxide (O_2_^•−^) and hydroxyl radicals (HO^•^).^[^ ^2^ ^]^ However, most of the PSs reported previously or used in clinics are Type‐II PSs, which are heavily O_2_‐dependent and, thus less effective in treating hypoxic solid tumors. In contrast, the PDT activity of Type‐I PSs is less O_2_‐dependent due to the partial O_2_‐recycling mode of action.^[^ ^3^ ^]^ Moreover, Type‐I PSs could intracellularly induce the production of the most cytotoxic HO^•^ by the superoxide dismutase (SOD)‐mediated disproportionation reaction of O_2_^•−^ (to produce hydrogen peroxide (H_2_O_2_)) and subsequent Fenton/Haber‐Weiss reactions of H_2_O_2_ (to produce HO^•^),^[^ ^4^ ^]^ thereby showing better PDT performance in treating hypoxic solid tumors. However, lacking a well‐defined design strategy, only a few Type‐I PSs have been reported to date, mainly including metal complexes and conjugated organic molecules.^[^ ^4^, ^5^ ^]^ The metal complex‐based Type‐I PSs, although effective in producing O_2_^•−^ or HO^•^, commonly exhibit short excitation wavelengths in the visible region, thus disadvantageous for treating the deep‐seated tumor tissue, which, coupled with the potential dark cytotoxicity of metal ions, partially limits their potential for application in clinical practice. Indeed, the concern has greatly motivated the interest to develop more biocompatible organic Type‐I PSs, typically including benzophenothiazine, BIDIPY trimer, and triphenylamine‐fluorene‐naphthalimide conjugator.^[^ ^4^, ^5^ ^]^ Even so, it remains a challenge to achieve the organic Type‐I PS that simultaneously possesses the high photon‐capturing ability, efficient O_2_^•−^ generation, precise tumor targeting, and biosafe renal metabolism. To this end, a well‐defined ISC mechanism must first be identified for a selected organic chromophore to endow it with excellent photosensitivity.

**Scheme 1:**
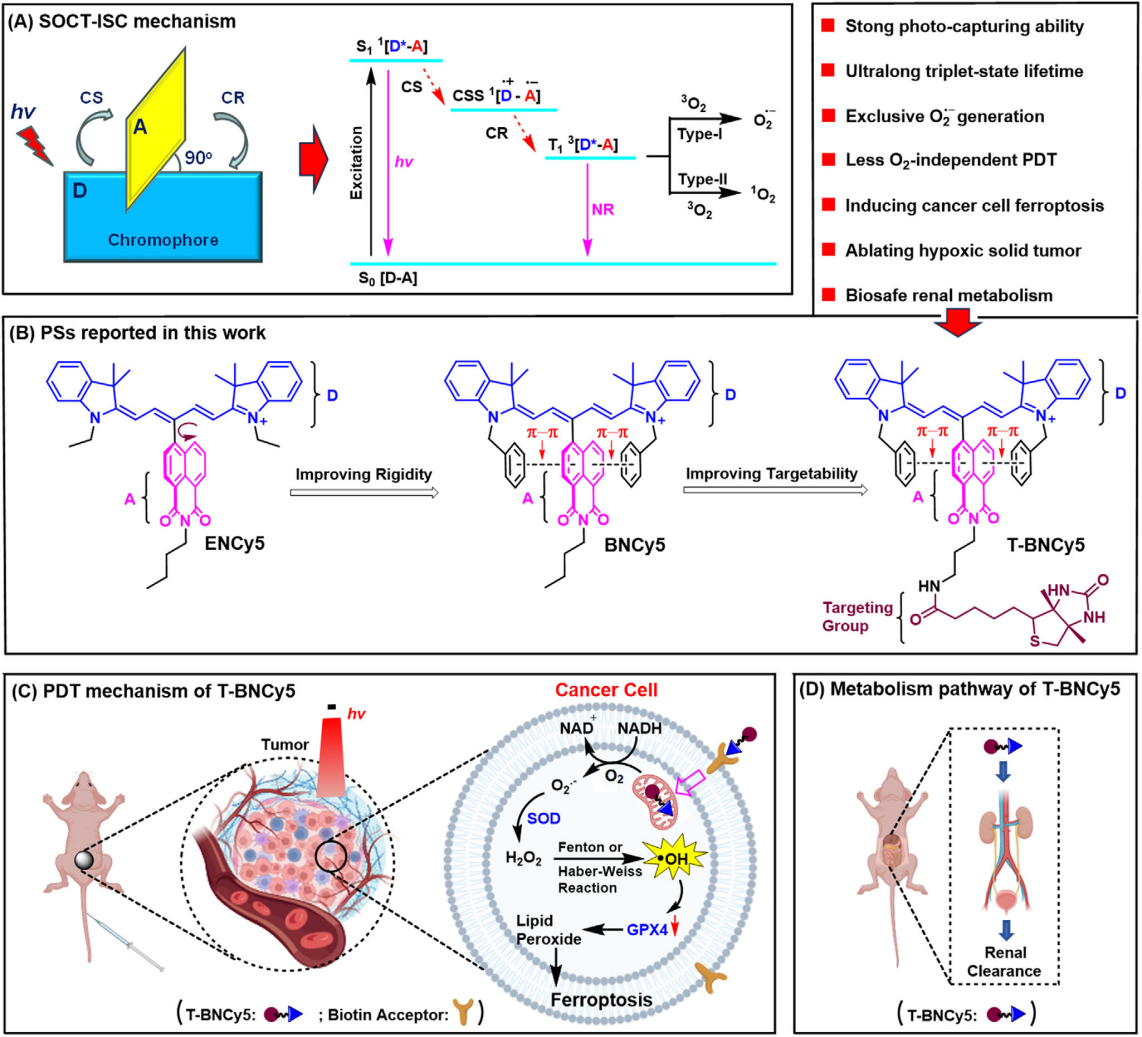
A) Schematic representation of the orthogonal D−A molecular structure for the SOCT‐ISC‐based PSs as well as the simplified Jablonski diagram illustrating the paths for T1‐state population and ROS generation via Type‐I or Type‐II mechanism. B) Chemical structures of **ENCy5**, **BNCy5**, and **T‐BNCy5** reported in this work, where π–π stacking was indicated. C) The PDT mechanism of **T‐BNCy5** and D) its in vivo metabolism via renal clearance.

Among various ISC mechanisms, the spin‐orbit charge transfer intersystem crossing (SOCT‐ISC) has achieved considerable success in constructing the heavy‐atom‐free organic PS,^[^ ^6^ ^]^ and the corresponding PS is composed of a closely linked electron donor (D) and acceptor (A) dyad, in which the π systems of D and A are required to be conjugatively uncoupled and sterically orthogonal for ensuring an effective SOCT‐ISC process (**Scheme** **1**A). The photophysics of the mechanism involves a photoinduced charge separation (CS) to form a charge separation state (CSS) and subsequent charge recombination (CR) to populate the T_1_ state.

**Figure 1:**
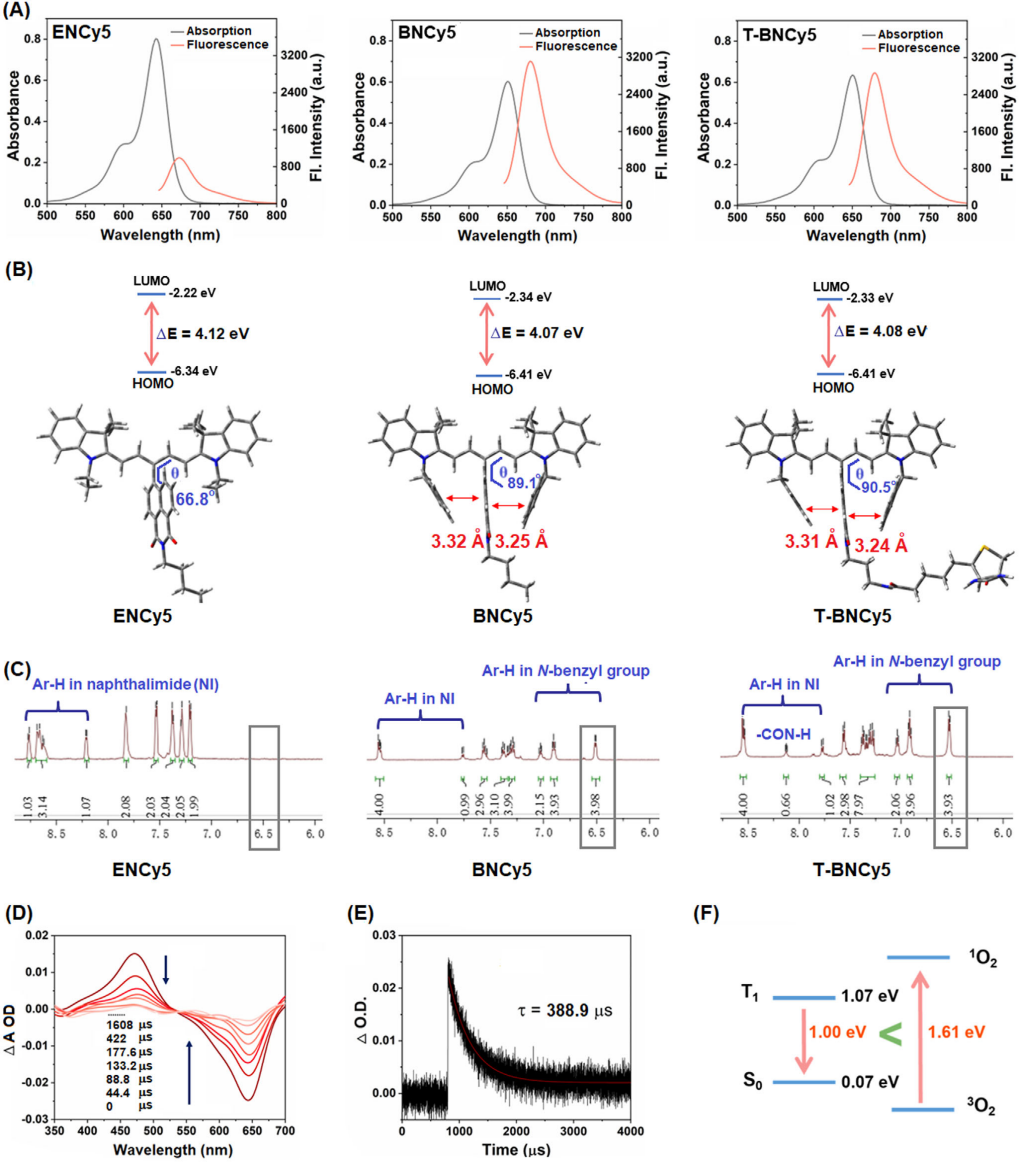
A) Absorption and fluorescence spectra of **ENCy5**, **BNCy5**, and **T‐BNCy5** (all 4 µm) in DMSO. B) The calculated HOMO and LUMO energy levels and optimized molecular structures of **ENCy5**, **BNCy5,** and **T‐BNCy5** at m062x/6‐31g* level. C) The ^1^H‐NMR signals of **ENCy5**, **BNCy5,** and **T‐BNCy5** in the aromatic area. D) The nanosecond transient absorption spectra of **T‐BNCy5** (λ_ex_ = 532 nm) and E) the decay kinetics of ESA signals at 460 nm. F) The calculated T_1_‐S_0_ energy gaps of **T‐BNCy5** and ^1^O_2_ ‐^3^O_2_, respectively.

Although attractive, the previously reported SOCT‐ISC‐based PSs suffer from several limitations when considering future applications in clinic: 1) none of them is of pure Type‐I PS, thus less effective in treating hypoxic solid tumors; 2) their D‐A geometries, albeit sterically orthogonal, are not fully rigid, thus detrimental to the occurrence of an efficient SOCT‐ISC;^[^ ^6d^ ^]^ 3) nearly all of them lack an appropriate handle for introducing a tumor‐targeting unit, thus disadvantageous to the precise PDT of tumors. In addition, although improving water solubility can shift the metabolic pathway from liver excretion to the more desirable renal clearance,^[^ ^7^ ^]^ many SOCT‐ISC‐based PSs lose their photosensitivity in water due to the largely decreased energy level of CSS state in the highly polar solvent effectively promoting its nonradiative decay to S_0_ state instead of ISC to T_1_ state.^[^ ^8^ ^]^ Overall, developing the new SOCT‐ISC‐based PSs capable of overcoming all the above limitations is urgently demanded.

In this work, a new SOCT‐ISC‐based PS, i.e., **T‐BNCy5** (Scheme 1B), was presented by integrating a biotin‐functionalized naphthalimide moiety (acting as A) into the *meso*‐position of a strongly photon‐capturing, *N*‐benzyl‐functionalized pentamethine cyanine (Cy5) dye (acting as D), which exclusively produced O_2_^•−^ via Type‐I pathway under the low‐power 660 nm photoirradiation. Such molecular engineering endowed **T‐BNCy5** with a near‐perfect D‐A orthogonal geometry and strong structural rigidity by forming an intracellular sandwich‐like π–π stacking assembly between *N*‐benzyl and naphthalimide units, which not only facilitates SOCT‐ISC to populate an ultralong‐lifetime T_1_ state (up to 389 µs) but also largely improves the O_2_^•−^‐generating ability. In vitro assays revealed that **T‐BNCy5** could specifically accumulate in mitochondria due to its positively charged Cy5 unit, where it not only generated O_2_^•−^ under photoirradiation but also induced the burst of the most cytotoxic HO^•^ by a cascade of biochemical reactions including the SOD‐mediated disproportionation and subsequent Fenton/Haber‐Weiss reactions (Scheme 1C), thereby severely damaging mitochondria and triggering cell ferroptosis. Whether under normoxia or hypoxia, **T‐BNCy5** showed a strong cancer cell‐killing ability under photoirradiation with the half maximal inhibitory concentration (IC_50_) value ≈0.45 µm. In vivo assays in mouse models showed that **T‐BNCy5** preferentially accumulated at the tumor site post intravenous injection, and after a single PDT treatment, it not only ablated tumors almost completely but also could rapidly be cleared from the body through the biosafe urinary excretion (Scheme 1D), indicative of its great potential for application in future clinical practice.

## 2 Results and Discussion

### 2.1 Molecular Design and Synthesis

To achieve an efficient SOCT‐ISC‐based PS, we chose pentamethine cyanine (Cy5) as an electron donor due to its large molar extinction coefficient (> 10^5^ m ^−1^ cm^−1^) and long absorption and emission wavelengths (≈660 nm).^[^ ^9^ ^]^ As for electron acceptor, we selected the moderately electron‐deficient naphthalimide group [NI, (E_Red_ = −1.85 V, Fc/Fc^+^)] with an expectation that the resulting PS would have an elevated CSS‐state energy level in the highly polar water, thereby avoiding the loss of its photosensitivity via CSS→S_0_ non‐radiative decay.^[^ ^6^, ^10^ ^]^ In addition, naphthalimide group enjoys the merit of easy modification to introduce an alkylamino handle at its imide N atom, thus providing an opportunity for constructing the tumor‐targeted PS by attaching a tumor‐targeting group to the handle. As a proof of concept, we chose the widely used biotin ligand as the tumor‐targeting group, given that many types of cancer cells overexpress the high‐affinity biotin transporter on their membrane surface.^[^ ^11^ ^]^ Further, to ensure a rigid orthogonal geometry between naphthalimide unit and the Cy5 polyene chain to augment the SOCT‐ISC efficiency,^[^ ^12^ ^]^ the benzyl group was selected to be attached at two terminal indolenium N sites of Cy5 skeleton in a hope that the two *N*‐benzyl groups could form π–π interaction with *meso*‐naphthalimide unit from its both sides, thereby improving the orthogonality of the resulting PS (Scheme 1B). With these considerations in mind, we designed **BNCy5** and its tumor‐targeted version **T‐BNCy5** by covalently installing the corresponding naphthalimide electron acceptor to the *meso*‐position of a *N*‐benzyl‐functionalized Cy5 dye, respectively. For comparison, a *N*‐ethyl‐functionalized Cy5‐naphthalimide dyad **ENCy5** was also designed. To our delight, the theoretical calculation (with **T‐BNCy5** as a representative) revealed a small T_1_−S_0_ energy gap of 1.00 eV, which is much smaller than the ^3^O_2_−^1^O_2_ energy gap (1.610 eV), indicating that it is less possible for the three dyads to undergo a Type‐II pathway to produce ^1^O_2_ (see below).^[^ ^13^ ^]^ That is to say, if the three dyads have the potential to produce ROS under photoirradiation, it is most likely O_2_^•−^ produced via the Type‐I pathway. To test the possibility, we prepared **ENCy5** and **BNCy5** by the Suzuki‐Miyaura coupling reactions between a naphthalimide‐based boronic acid ester and corresponding *meso*‐Br substituted Cy5 dye, respectively. **T‐BNCy5** was synthesized by a standard peptide bond‐forming reaction between biotin and an amino precursor of **T‐BNCy5**, i.e., **A‐BNCy5** (Supporting Information) that was pre‐prepared by a similar procedure as **ENCy5** or **BNCy5**. Synthetic procedures and characterization data including ¹H‐NMR, ¹^3^C‐NMR, and HRMS spectra were provided in Supporting Information.

### 2.2 Photophysical Properties and Theoretical Calculations

With **ENCy5**, **BNCy5**, and **T‐BNCy5** in hand, we first studied their photophysical properties. The absorption and emission spectra in DMSO are shown in **Figure** **1**A and those in water are shown in Figure S1 (Supporting Information), and the corresponding photophysical data are summarized in **Table** **1**. As seen, all three dyes show the typical absorption and emission bands of Cy5 dye peaked at ca. 650 and 670 nm, respectively, indicative of the negligible electronic coupling between their *meso*‐naphthalimide and Cy5 units. Notably, the absorption and emission wavelengths of **BNCy5** and **T‐BNCy5** are slightly longer than those of **ENCy5**, consistent with the theoretical calculation that revealed a slightly narrower HOMO‐LUMO energy gap for the former two than the latter (Figure 1B). The molar extinction coefficients of the three dyes are all above 1.3 × 10^5^ m ^−1^ cm^−1^ whether in DMSO or water, revealing their strong photon‐capturing abilities. Moreover, all of them show good water solubility of at least up to 20 µm, as evidenced by the concentration‐dependent absorption spectra changes that match well with the Lambert‐Beer law (Figure S2, Supporting Information). However, compared with conventional Cy5 dye,^[^ ^9a^ ^]^ their fluorescence quantum yields (Φ_f_) are smaller, especially in water, indicative of the existence of a photoinduced charge separation process that populates a nonfluorescent CSS state.^[^ ^14^ ^]^ The higher Φ_f_ values of **BNCy5** and **T‐BNCy5** than that of **ENCy5** also indicate that the former two, featured with two *N*‐benzyl groups in their Cy5 skeleton, have larger structural rigidity than the latter that partially inhibits their non‐radiative decay.

**Table 1:** Photophysical properties of Cy5 derivatives.

PSs	Solvent	λ_abs_/λ_em_ [nm]	ε [M^−1^ cm^−1^]	Φ_f_ [%]^a)^
Cy5^b)^	DMSO	655/668	2.72 × 10^5^	33.0
	Water	648/656	2.27 × 10^5^	13.0
ENCy5	DMSO	642/673	2.01× 10^5^	7.6
	Water	634/668	1.74 × 10^5^	3.4
BNCy5	DMSO	650/680	1.50 × 10^5^	16.2
	Water	644/670	1.33 × 10^5^	4.2
T‐BNCy5	DMSO	650/679	1.59 × 10^5^	13.5
	Water	646/672	1.34 × 10^5^	5.0

^a)^ Quantum yields determined using Cy5 (Φ_f_ = 0.15 in MeOH) as reference.

^b)^ The photophysical properties of Cy5 were obtained from the reported data.^[^ ^17^ ^]^

It is worth noting that the rigid orthogonal geometry between electron donor and acceptor is necessary for the efficient occurrence of SOCT‐ISC to populate the T_1_ state.^[^ ^12^ ^]^ Thus, the DFT‐optimized molecular structures of the three dyads were analyzed. As shown in Figure 1B, for **BNCy5** or **T‐BNCy5**, the *meso*‐naphthalimide unit and Cy5 unit were found to be almost orthogonal to each other with dihedral angles (θ) calculated to be 89.1° and 90.5°, respectively; by comparison, a smaller dihedral angle of 66.8° was observed for **ENCy5**. Importantly, it was found that for **BNCy5** and **T‐BNCy5**, several phenyl C‐atoms in each *N*‐benzyl group are spatially very close to the *meso*‐naphthalimide plane from both sides with the shortest distance of ≈3.3 Å, strongly indicative of the formation of a π–π stacking assembly between them.^[^ ^15^ ^]^ The formation of such π–π stacking could be supported by the ^1^H‐NMR studies of **BNCy5** or **T‐BNCy5**, where two aromatic protons in each *N*‐benzyl unit surprisingly display obvious upfield shift (up to 6.5 ppm) relative to other aromatic protons in the *N*‐benzyl unit probably due to the ring current effect of the aromatic *meso*‐naphthalimide unit (Figure 1C); in contrast, the case was not observed in **ENCy5** that lacks such *N*‐benzyl unit. Further, an ≈10 nm red‐shift in the absorption and emission spectra of **BNCy5** or **T‐BNCy5** relative to those of **ENCy5** also supports the presence of such π–π stacking (Table 1), considering that the electron‐rich benzene unit in *N*‐benzyl group could decrease the electron‐withdrawing ability of *meso*‐naphthalimide unit by π–π interaction, thus red‐shifting the absorption and emission wavelengths compared to those of **ENCy5** in terms of the substituent effects of *meso*‐groups for Cy5 (Figure 1B).^[^ ^16^ ^]^ The decreased electron‐withdrawing ability of the *meso*‐naphthalimide unit in **BNCy5** or **T‐BNCy5** by such π–π interaction could be supported by the obvious upfield shifts of its aromatic protons relative to those in **ENCy5** (Figure 1C). In addition, the higher Φ_f_ values of **BNCy5** (0.16) and **T‐BNCy5** (0.14) than that of **ENCy5** (0.08) in DMSO also support the presence of such π–π stacking in the former two, which induces larger structural rigidity and thus improves their Φ_f_ values. As a result, the two *N*‐benzyl groups in **BNCy5** or **T‐BNCy5** behave more like two “crab pincers” that tightly hold the *meso*‐naphthalimide unit by the π–π stacking interaction, thus limiting its torsion motion relative to Cy5 polyene chain and ensuring its orthogonality with Cy5 chromophore (Figure 1B). In this sense, **BNCy5** and **T‐BNCy5** should enjoy stronger abilities than **ENCy5** to populate the T_1_ state in terms of the SOCT‐ISC mechanism.

To explore the SOCT‐ISC mechanism, we evaluated the photoinduced charge separation to form a CSS state by DFT calculation with **T‐BNCy5** as a representative. As shown in Figure S3 (Supporting Information), based on the 0.6 eV guideline developed by the Liu group,^[^ ^18^ ^]^ the minimal molecular orbital energy gap (ΔE) between chromophore (Cy5 unit) and quencher (*meso*‐naphthalimide unit) was calculated to be 0.56 eV, indicating that **T‐BNCy5** can undergo a charge separation to populate the CSS state upon photoexcitation. Further, we performed an electrochemical study of **T‐BNCy5** using cyclic voltammetry to explore the thermodynamic feasibility of the charge separation process. By determining the reduction and oxidation potentials of **T‐BNCy5** (‐1.57 and 0.13 eV, respectively), the Gibbs free energy change (ΔG_PeT_) of the charge separation was calculated to be −0.28 eV according to the Rehm‐weller equation (Figure S4, Supporting Information), indicating that the charge separation of **T‐BNCy5** under light excitation is thermodynamically allowed.^[^ ^19^ ^]^ The formation of the T_1_ state of **T‐BNCy5** could be confirmed experimentally by the nanosecond transient absorption spectra study.^[^ ^20^ ^]^ As shown in Figure 1D, the pulse laser excitation at 532 nm resulted in an excited state absorption (ESA) band (T_1_→T_n_ transition) at 460 nm and a ground state bleaching (GSB) band at 650 nm. The synchronous rise and decay of GSB and ESA bands generated a well‐defined isosbestic point, indicating that the T_1_‐state decay exclusively repopulates the S_0_ state. By monitoring the decay trace of the ESA band, the T_1_‐state lifetime of **T‐BNCy5** was determined to be as long as 388.9 µs (Figure 1E). Such an ultralong‐lived T_1_ state could be ascribed to its rigid orthogonal conformation, strongly indicating that **T‐BNCy5** should be an excellent PS that is capable of producing ROS via either an energy (Type‐II) or electron (Type‐I) transfer process under photoirradiation. Further, we conducted the triplet‐state calculation using the TD‐DFT method by setting the spin as a triplet. To our delight, the calculation gave rise to a T_1_‐S_0_ energy gap of 1.00 eV that is smaller than that of ^3^O_2_‐^1^O_2_ (1.61 eV) (Figure 1F),^[^ ^13^ ^]^ manifesting that the T_1_ state of **T‐BNCy5** is energetically insufficient to produce ^1^O_2_ via Type‐II mechanism. Thus, if **T‐BNCy5** would produce ROS under photoirradiation, it is most likely O_2_^•−^ generated via the Type‐I process.

### 2.3 ROS Evaluation under Photoirradiation

**Figure 2:**
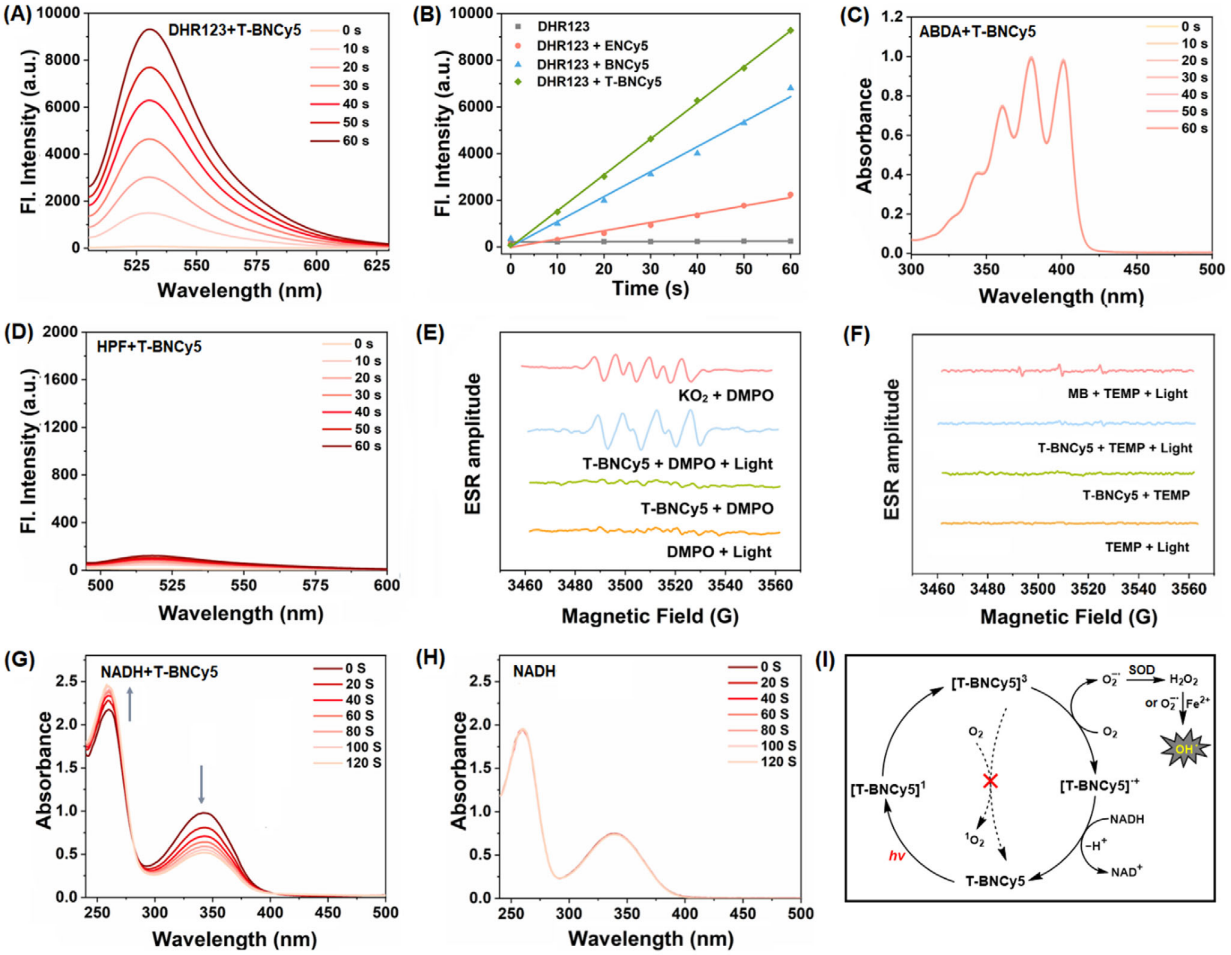
A) Fluorescence spectra change of the water solution of **T‐BNCy5**/DHR123 under photoirradiation (LED light, 660 nm, 20 mW cm^−2^). B) Fluorescence intensity changes of DHR123 at 529 nm in the absence and presence of **ENCy5**, **BNCy5,** and **T‐BNCy5**, respectively, under photoirradiation. C) Absorption spectra change of the PBS solution of **T‐BNCy5**/ABDA under photoirradiation. D) Fluorescence spectra change of the water solution of **T‐BNCy5**/HPF under photoirradiation. E) EPR spectra of DMPO under different conditions with KO_2_ as a reference (G values for the signals of KO_2_/DMPO from left to right: 3489, 3498, 3503, 3511, 3517, and 3524; and G values for the signals of **T‐BNCy5**/DMPO/light: 3489, 3498, 3502, 3512, 3516, and 3525). F) EPR spectra of TEMP under different conditions with methylene blue (MB) as the reference (G values for the signals of MB/TEMP/light from left to right: 3491, 3507, and 3523). G, H) Absorption spectra change of the aqueous solution of NADH in the presence and absence of **T‐BNCy5** under photoirradiation, respectively. I) Schematic illustration of the O_2_‐ and NADH‐mediated photocatalytic recycling of **T‐BNCy5** within cells that produces both O_2_^•−^ and HO^•^.

Encouraged by the above results, we subsequently evaluated the ROS generation abilities of **ENCy5**, **BNCy5,** and **T‐BNCy5** in an aqueous solution. First, we tested their O_2_^•−^‐generating abilities by using dihydrorhodamine 123 (DHR123, a commercial O_2_^•−^ probe).^[^ ^21^ ^]^ To our delight, upon photoirradiation of the mixed DHR123/**T‐BNCy5** solution using an LED light source (660 nm, 20 mW cm^−2^) for 60 s, a ≈128‐fold fluorescence enhancement of DHR123 at 529 nm was observed (**Figure** **2**A), indicating that **T‐BNCy5** could generate a substantial amount of O_2_^•−^ via the Type‐I process. A similar case was also observed for **BNCy5**, albeit with a slightly decreased O_2_^•−^‐generating ability compared to **T‐BNCy5**. In contrast, under the otherwise identical condition, the fluorescence enhancement of DHR123 in the presence of **ENCy5** was not obvious, indicative of its poor O_2_^•−^‐generating ability (Figure 2B; Figure S5, Supporting Information). The stronger O_2_^•−^‐generating abilities of **T‐BNCy5** and **BNCy5** than that of **ENCy5** under photoirradiation could be ascribed to their more rigid orthogonal geometries that not only facilitate the T_1_‐state population via SOCT‐ISC but also enhance T_1_‐state lifetime by inhibiting the non‐radiative decay. Further, we tested their ^1^O_2_‐ and HO^•^‐generating abilities by using 9′,10′‐anthracenediyl‐bis‐(methylene)‐dimalonic acid (ABDA, a commercial ^1^O_2_ probe) and hydroxyphenyl fluorescein (HPF, a commercial HO^•^ probe), respectively.^[^ ^22^ ^]^ Indeed, the ^1^O_2_ or HO^•^ generation of the three dyads was almost negligible under photoirradiation, as indicated by the nearly unchanged absorption and fluorescence spectra of ABDA and HPF (Figure 2C,D; Figures S6,S7, Supporting Information). To further confirm the abilities of the three dyads to exclusively produce O_2_^•−^ under photoirradiation, we performed an electron paramagnetic resonance (EPR) spectroscopy study with **T‐BNCy5** as a representative. In the assay, 5,5‐dimethyl‐1‐pyrroline‐N‐oxide (DMPO) and 2,2,6,6‐tetramethyl‐4‐piperidone hydrochloride (TEMP) were employed as spin probes to capture O_2_^•−^ and ^1^O_2_, respectively.^[^ ^5^, ^23^ ^]^ As shown in Figure 2E, F, upon photoirradiation of the mixed **T‐BNCy5**/DMPO solution, a characteristic paramagnetic signal of the DMPO‐O_2_^•−^ adduct appeared; conversely, no TEMP‐^1^O_2_ signal was detected for the mixed **T‐BNCy5**/TEMP solution after photoirradiation. Together, the above assays indicate that **ENCy5**, **BNCy5**, and **T‐BNCy5** all are of Type‐I PSs, but with **T‐BNCy5** behaving best in generating O_2_^•−^. Thus, **T‐BNCy5** was selected for subsequent studies.

It should be mentioned that, during the O_2_^•−^‐generating process, the T_1_‐state species would lose an electron to form a radical cation, which must get an electron from a reduced substrate to complete the photocatalytic recycling.^[^ ^3^ ^]^ Given that NADH (the reduced form of nicotinamide adenine dinucleotide) exists abundantly within cells and constitutes a cellular electron pool,^[^ ^24^ ^]^ we further evaluated whether NADH could act as a substrate to provide an electron to the produced radical cation. As shown in Figure 2G, upon the photoirradiation of the mixed NADH/**T‐BNCy5** solution, a dramatic decrease in absorbance at ≈340 nm (a characteristic peak of NADH) was observed, which, coupled with the fact that the absorption spectra of NADH itself remained stable under photoirradiation (Figure 2H), strongly indicates that NADH could act as an electron donor for the radical cation of **T‐BNCy5**. Given that **T‐BNCy5** is a mitochondria‐targeted PS (see below) and that NADH is a main electron donor of the mitochondria respiratory chain, it was expected that the PS could cause serious mitochondrial injury by generating both O_2_^•−^ (via the photocatalytic recycling) and HO^•^ (via a cascade of biochemical reactions) (Figure 2I).

### 2.4 In Vitro PDT Evaluation under Normoxia and Hypoxia

**Figure 3:**
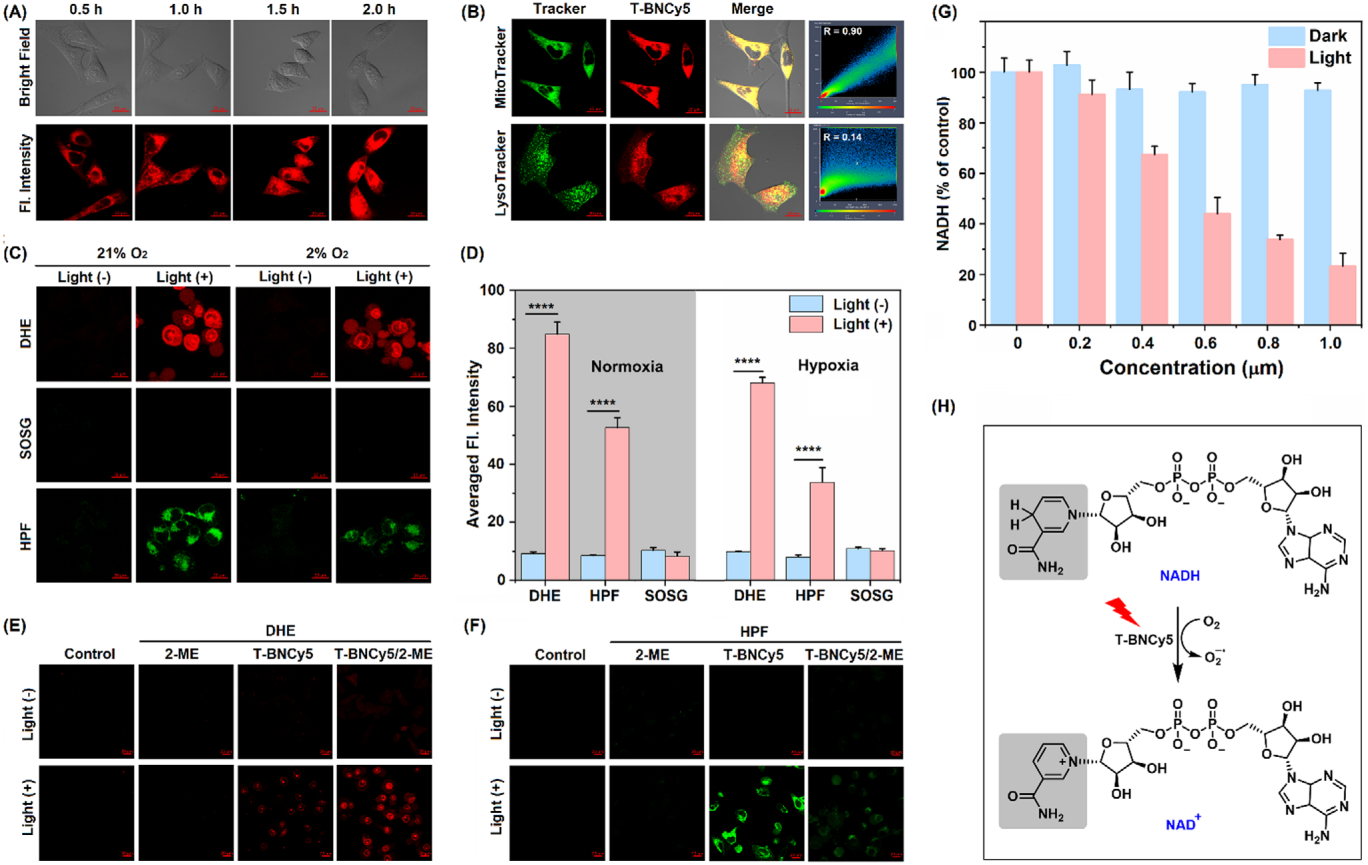
A) Time‐dependent cellular uptake evaluation of **T‐BNCy5** in MCF‐7 cells. B) Confocal images of MCF‐7 cells co‐stained with **T‐BNCy5**/Mito Tracker Green FM or **T‐BNCy5**/Lyso Tracker® Green. R refers to the Pearson correlation coefficient. C) ROS detection in MCF‐7 cells was performed under both normoxic (21% O_2_) and hypoxic (2% O_2_) conditions using DHE, HPF, and SOSG, respectively. D) The quantitative representation of the fluorescence intensities in (C). E) Confocal fluorescence images of MCF‐7 cells co‐stained with **T‐BNCy5**/DHE/2‐Me with and without photoirradiation and corresponding control images. F) Confocal fluorescence images of MCF‐7 cells co‐stained with **T‐BNCy5**/HPF/2‐Me with and without photoirradiation and corresponding control images. G) Quantitative analysis of intracellular NADH level in MCF‐7 cells pretreated with the varied concentration of **T‐BNCy5** and then treated with and without photoirradiation. H) Schematic representation of the photocatalytic NADH→NAD^+^ transformation. Irradiation condition: 660 nm LED light source, 20 mW cm^−2^, 20 min. For DHE, λ_ex_/λ_em_ = 488/560‐660 nm; for HPF, λ_ex_/λ_em_ = 488/493‐600 nm; for SOSG, λ_ex_/λ_em_ = 488/493‐565 nm. Scale bar in A–C,E,F): 20 µm. *P < 0.05, **P < 0.01, ***P < 0.001, and ****P < 0.0001.

The in vitro PDT performances of **T‐BNCy5** were evaluated in cancerous MCF‐7 cells using a confocal laser‐scanning microscope (CLSM). Before these assays, we first studied the cell uptake and subcellular location of **T‐BNCy5**. As shown in **Figure** **3**A, based on the time‐dependent intracellular fluorescence changes, it was evident that **T‐BNCy5** could be internalized by MCF‐7 cells within 2 h. Moreover, the intracellular fluorescence of **T‐BNCy5** exhibited a high correlation with commercial Mitotracker with the Pearson's correlation coefficient (*R*) value up to 0.90; by comparison, **T‐BNCy5** showed a completely different subcellular distribution from commercial LysoTracker, ER‐Tracker, and Hoechst 33342, as reflected by the lower *R* values (Figure 3B; Figure S8, Supporting Information). Thus, **T‐BNCy5** is a mitochondria‐targeted PS, which could be ascribed to its positively charged Cy5 unit as well as the highly negative mitochondria membrane potentials.^[^ ^25^ ^]^ Mitochondria have broadly been recognized as one of the most ideal target organelles for PDT due to their great vulnerability to excessive ROS.^[^ ^26^ ^]^ In this sense, **T‐BNCy5** was greatly expected to be an outstanding PS for killing cancer cells under photoirradiation.

Subsequently, we assessed the ROS‐generating profile of **T‐BNCy5** in cancerous MCF‐7 cells under photoirradiation (660 nm LED light, 20 mW cm^−2^, 20 min) by CLSM. First, we tested the O_2_^•−^‐generating ability of **T‐BNCy5** in the cells using a commercial O_2_^•−^ probe dihydroethidium (DHE).^[^ ^6^, ^27^ ^]^ As shown in Figure 3C, D, under normoxia (21% O_2_), the photoirradiation of the **T‐BNCy5/**DHE co‐stained MCF‐7 cells led to a 9‐fold fluorescence enhancement in DHE channel relative to that without photoirradiation, indicative of the strong O_2_^•−^‐generating ability of the PS within cells. Even under hypoxia (2% O_2_), the photoirradiation also resulted in an obvious fluorescence enhancement of up to 7‐fold in the DHE channel, suggesting that **T‐BNCy5** also has a strong O_2_^•−^‐generating ability under hypoxia. Further, using a commercial ^1^O_2_ probe SOSG, we found that whether under normoxia or hypoxia, the photoirradiation of the **T‐BNCy5/**SOSG co‐stained MCF‐7 cells did not produce ^1^O_2_, consistent with the case found in the aqueous solution, further confirming the role of **T‐BNCy5** as a Type‐I PS. Also, the HO^•^‐generating ability of **T‐BNCy5** was evaluated using a commercial HO^•^ probe HPF.^[^ ^22b^ ^]^ Upon photoirradiation of the **T‐BNCy5**/HPF co‐stained MCF‐7 cells, an obvious fluorescence enhancement relative to that without photoirradiation was observed in the HPF channel under either normoxia or hypoxia, indicative of the generation of HO^•^ within the cells. The result is distinct from that found in aqueous solution and could be attributed to the SOD‐mediated disproportionation of O_2_^•−^ (to produce H_2_O_2_) and subsequent Fenton and Haber‐Weiss reactions (to produce HO^•^).^[^ ^4^, ^28^ ^]^ To confirm the mechanism, we evaluated the concentration change of O_2_^•−^ or HO^•^ within MCF‐7 cells in the absence and presence of 2‐methoxy estradiol (2‐ME, a potent SOD inhibitor),^[^ ^4^ ^]^ respectively. As shown in Figure 3E, the DHE/2‐ME co‐stained MCF‐7 cells with and without photoirradiation showed unchanged fluorescence intensity compared to the control group, eliminating the possible interference of endogenous O_2_^•−^. When MCF‐7 cells were co‐stained with **T‐BNCy5**/DHE in the absence and presence of 2‐ME, respectively, and then subjected to photoirradiation, the latter showed brighter fluorescence than the former in the DHE channel, consistent with the strong inhibition action of 2‐ME to SOD that prevents O_2_^•−^ consumption. Using a HO^•^ probe HPF instead of DHE, we also evaluated the concentration change of HO^•^ in the otherwise identical condition. As expected, the fluorescence in **T‐BNCy5**/HPF/2‐ME group in the HPF channel almost diminished after photoirradiation, distinct from the bright fluorescence found in **T‐BNCy5**/HPF group (Figure 3F), indicating that the HO^•^‐generating ability of **T‐BNCy5** largely relies on the SOD activity and intracellular O_2_^•−^ content. In addition, we also tested the intracellular NADH level change with a commercial NADH kit after the **T‐BNCy5**‐loaded MCF‐7 cells were irradiated by the same LED light. As shown in Figure 3G, with the concentration of **T‐BNCy5** increasing, the intracellular NADH level markedly decreased, down to 20% of the initial level when 1 µm **T‐BNCy5** was used, strongly indicating that NADH could act as an endogenous electron source to participate in the photocatalytic recycling. Given that HO^•^ is the most reactive ROS in cells, **T‐BNCy5** was largely expected to be an effective PS for killing cancer cells.

**Figure 4:**
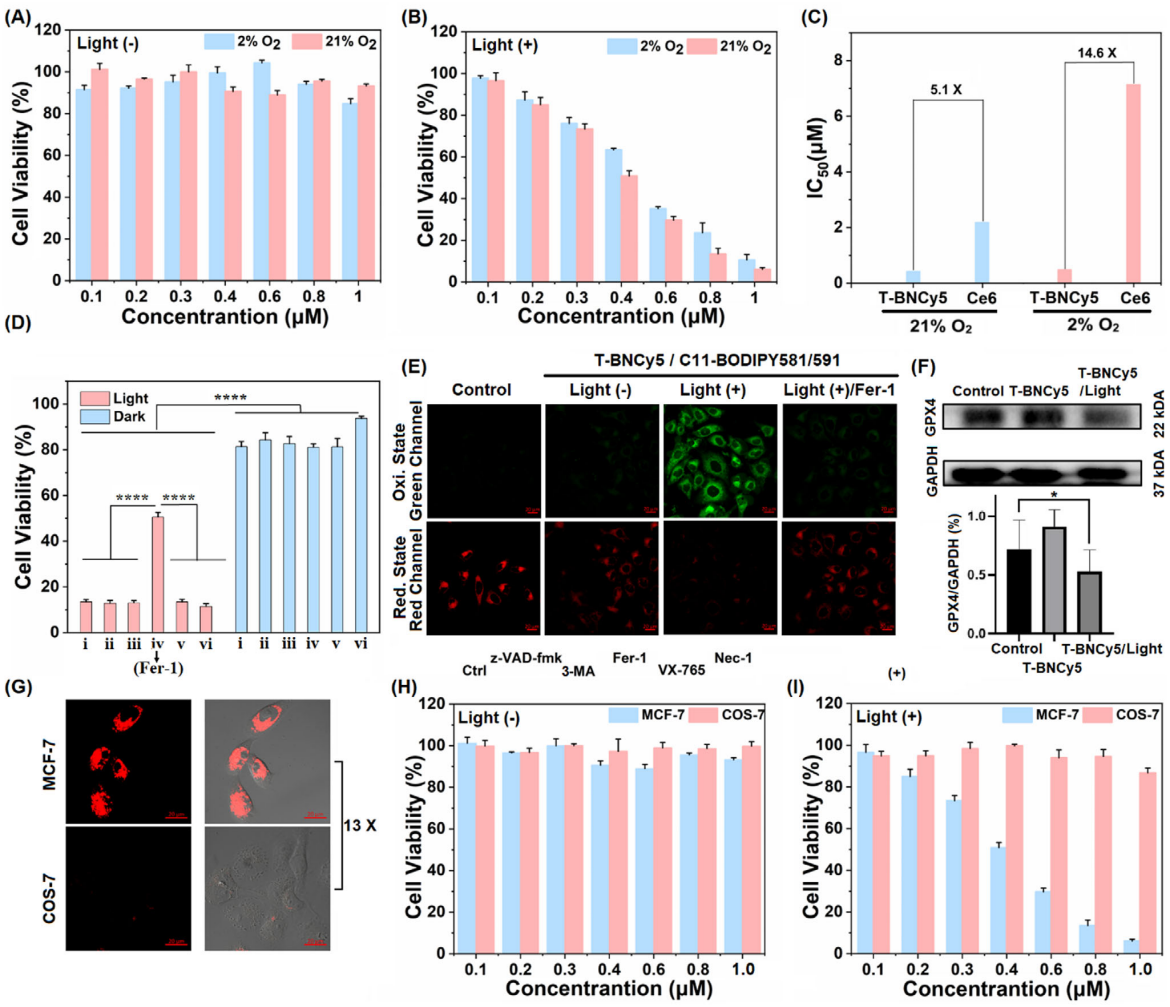
A, B) The dose‐dependent viability of MCF‐7 cells incubated with **T‐BNCy5** and then treated without and with 660 nm LED light (20 mW cm^−2^, 20 min) in normoxia (21% O_2_) and hypoxia (2% O_2_), respectively. C) A comparison of the IC_50_ values of **T‐BNCy5** and Ce6 for MCF‐7 cells under normoxia and hypoxia. D) Cell viability of the **T‐BNCy5**‐loaded MCF‐7cells pretreated with various cell death inhibitors (i: Ctrl; ii: z‐VAD‐fmk; iii: 3‐MA; iv: Fer‐1; v: VX‐765; vi: Nec‐1) and then treated with and without 660 nm LED photoirradiation. E) Confocal images of MCF‐7 cells co‐stained with **T‐BNCy5**/C11‐BODIPY 581/591 in the absence and presence of Fer‐1 and then treated with and without photoirradiation. For green channel, λ_ex_/λ_em_ = 488/505‐550 nm, and for red channel, λ_ex_/λ_em_ = 561/580‐640 nm. F) Western blot analysis of the expression of GPX4 in MCF‐7 cells after the different treatments. G) Confocal images of MCF‐7 and COS‐7 cells co‐stained with **T‐BNCy5** for 2 h, respectively. For **T‐BNCy5** channel, λ_ex_/λ_em_ = 633/638‐740 nm. H, I) The dose‐dependent viability of MCF‐7 cells and COS‐7 cells incubated with **T‐BNCy5** and then treated without and with 660 nm LED light. Scale bar in (E) and (G): 20 µm. *P < 0.05, **P < 0.01, ***P < 0.001, and ****P < 0.0001.

Moving on, we evaluated the PDT efficacy of **T‐BNCy5** toward MCF‐7 cells by the methyl thiazolyltetrazolium (MTT) assays. As shown in **Figure** **4**A, **T‐BNCy5** with concentrations below 1 µm showed negligible dark cytotoxicity under both normoxia (21% O_2_) and hypoxia (2% O_2_), indicative of its good biocompatibility. Upon photoirradiation (660 nm LED light, 20 mW cm^−2^, 20 min), **T‐BNCy5**, under normoxia (21% O_2_), efficiently inhibited the cell proliferation in a dose‐dependent manner with the half‐maximal inhibitory concentration (IC_50_) value as low as 0.43 µm (Figure 4B). By comparison, the IC_50_ value of the clinical photosensitizer Ce6 (a Type‐II PS) was 2.2 µm under the otherwise identical condition, 5.1‐fold higher than that of **T‐BNCy5** (Figure 4C). Further, we tested the PDT efficacy of **T‐BNCy5** under hypoxia (2% O_2_). Similarly, the photoirradiation also resulted in high cytotoxicity for MCF‐7 cells with IC_50_ values up to 0.49 µm (Figure 4B); by comparison, Ce6 showed lower PDT activity in the condition with IC_50_ value as high as 7.14 µm (Figure 4C), 14.6‐fold higher than that of **T‐BNCy5**, highlighting the important role of **T‐BNCy5** as a less O_2_‐dependent Type‐I PS to effectively kill cancer cells. The strong cancer cell‐killing ability of **T‐BNCy5** was demonstrated by the live/dead cell co‐staining assay (Figure S9, Supporting Information), where calcein AM (green fluorescence) and propidium iodide (PI; red fluorescence) were used to mark live and dead cells, respectively. Overall, these results reveal that **T‐BNCy5** possesses a high PDT efficacy in killing cancer cells with IC_50_ values up to submicromolar level whether under normoxia or hypoxia.

To explore the cell death mechanism, we performed MTT assays of the **T‐BNCy5**‐loaded MCF‐7 cells pre‐treated with various cell death inhibitors, respectively, followed by photoirradiation. As shown in Figure 4D, no significant increase in cell viability was observed in the groups pre‐treated with 3‐methyladenine (3‐MA, autophagy inhibitor), necrostatin‐1 (Nec‐1, necroptosis inhibitor), belnacasan (VX‐765, pyroptosis inhibitor), and L‐alaninamide (z‐VAD‐fmk, apoptosis inhibitor), respectively, when compared with control group.^[^ ^21^, ^29^ ^]^ In contrast, the group pre‐treated with ferrostatin‐1 (Fer‐1, a commercial ferroptosis inhibitor) gave rise to a remarkable enhancement in cell viability, suggesting that ferroptosis is the primary cell death pathway. Given that lipid peroxidation (LPO) is an important feature of ferroptosis, we used C11‐BODIPY581/591 (a commercial LPO probe: red fluorescence refers to its reduction state, and green fluorescence to its oxidation state due to LPO) to monitor LPO in MCF‐7 cells.^[^ ^29^, ^30^ ^]^ As shown in Figure 4E, after 660 nm LED irradiation, MCF‐7 cells in **T‐BNCy5**/C11‐BODIPY group exhibited strong green fluorescence, whereas no such green fluorescence was recorded in the control group, light (‐) group, and **T‐BNCy5**/C11‐BODIPY/Fer‐1 group. The confocal imaging result is consistent with the flow cytometry assay result (Figure S10, Supporting Information), both of which strongly indicate the obvious LPO accumulation during the PDT process of **T‐BNCy5**. Additionally, the expression of glutathione peroxidase (GPX4, which catalyzes the reduction of LPO by GSH in cells) was also evaluated. By western blotting assays,^[^ ^21^, ^29^ ^]^ it was found that intracellular GPX4 level was downregulated in the **T‐BNCy5**/Light group relative to the **T‐BNCy5** group and control group (Figure 4F; Figure S11, Supporting Information), consistent with cell ferroptosis. The markedly downregulated GPX4 level in the **T‐BNCy5**/Light group was further supported by the immunofluorescence staining assay (Figure S12, Supporting Information). Also, using a mitochondrial membrane potential (Δ*Ψm*) probe JC‐1, we demonstrated the obvious loss of the mitochondrial membrane potential, as revealed by a marked red‐to‐green fluorescence transition in the MCF‐7 cells co‐stained with **T‐BNCy5**/JC‐1 and then treated under photoirradiation (Figure S13, Supporting Information).^[^ ^6m^ ^]^ Together, the above results confirm that the **T‐BNCy5**‐induced PDT efficiently induces LPO, downregulates GPX4 level, and damages mitochondria, ultimately triggering cancer cell ferroptosis.

Before in vivo studies, we compared the cellular uptake and killing abilities of **T‐BNCy5** toward cancerous and normal cells under CLSM, respectively. In the assays, the biotin receptor‐positive cancerous MCF‐7 cells and ‐negative normal COS‐7 cells were employed as representatives, respectively.^[^ ^11^ ^]^ After being treated with the same concentration of **T‐BNCy5** for 2 h, the integrated fluorescence intensity of MCF‐7 cells was found to be 13‐fold higher than that of COS‐7 cells, indicating that the appended biotin moiety endows **T‐BNCy5** with an excellent cancer cell‐targeting ability (Figure 4G). Encouraged by the result, the potential of **T‐BNCy5** in selectively killing cancer cells was tested by MTT assays. As shown in Figure 4H, I and **T‐BNCy5** below 1 µm showed negligible dark cytotoxicity toward the two cell lines; however, upon the LED photoirradiation (660 nm, 20 mW cm^−2^, 20 min), significant cell death in cancerous MCF‐7 cells was observed with the IC_50_ value as low as 0.43 µm. In contrast, normal COS‐7 cells survived the PDT treatment with cell viability > 80% even at a high **T‐BNCy5** concentration of 1.0 µm. These results manifest that the uptake and PDT performances of **T‐BNCy5** are highly selective for cancerous MCF‐7 cells over normal COS‐7 cells, indicating its great potential for the precise PDT of the biotin receptor‐positive tumors.

### 2.5 In Vivo Imaging and PDT Evaluation

**Figure 5:**
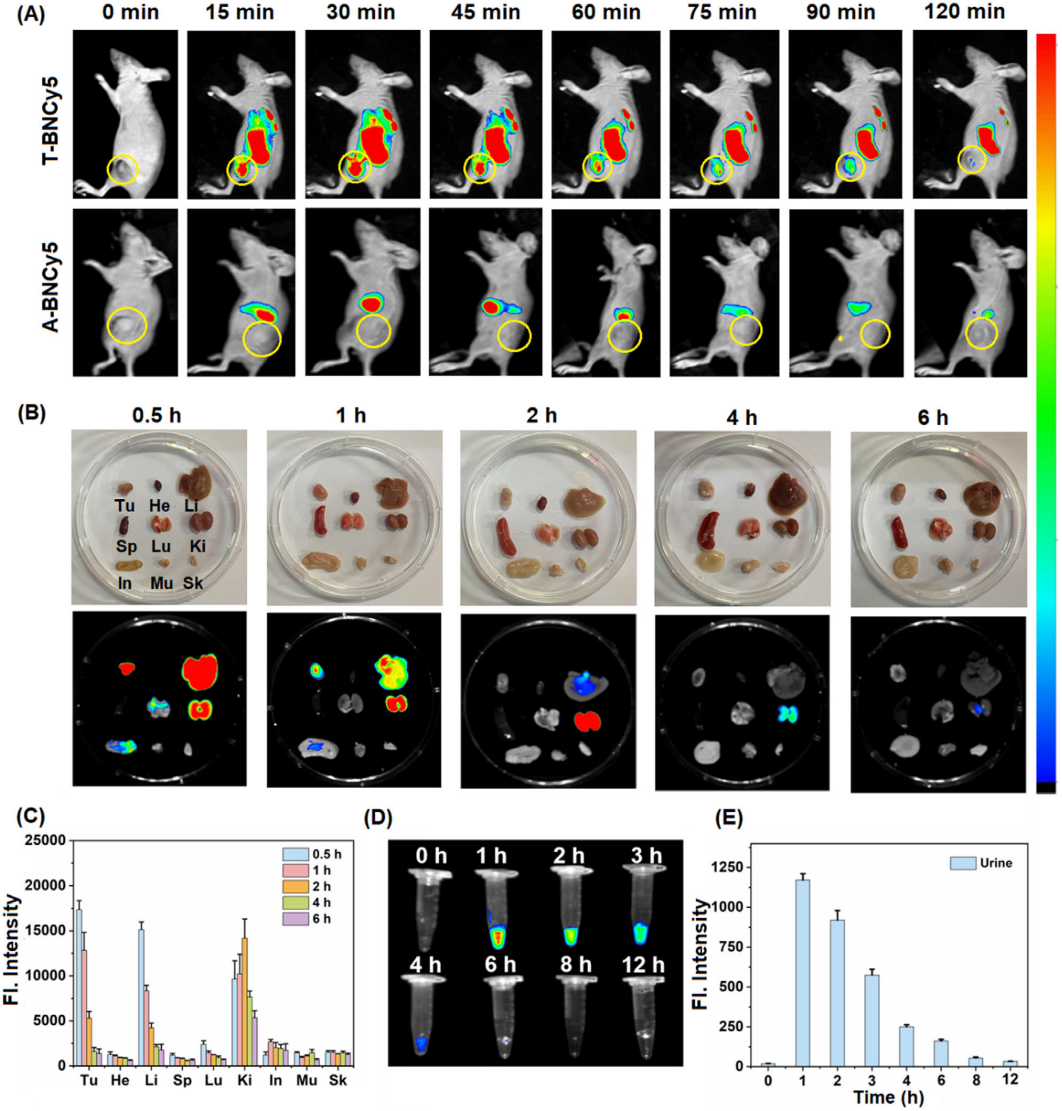
A) In vivo fluorescence imaging of the 4T1 tumor‐bearing mice at the indicated time points after intravenous injection of **T‐BNCy5** and **A‐BNCy5** (both, 100 µm, 100 µL), respectively. Note that, **A‐BNCy5** is a synthetic precursor of **T‐BNCy5**, which is in absence of biotin unit (Supporting Information). B) *Ex vivo* fluorescence images of the biodistribution of **T‐BNCy5** in tumor and major organs (three mice were used for each of time points, and only representative images were shown), including tumor (Tu), heart (He), liver (Li), spleen (Sp), lung (Lu), kidney (Ki), intestine (In), muscle (Mu), and skin (Sk), at 0.5, 1, 2, 4, and 6 h post intravenous injection, and C) the corresponding fluorescence intensity changes. D) Fluorescence images of urine at the indicated time points post intravenous injection of **T‐BNCy5** (a total of three mice were used, and only representative images were shown), and E) the corresponding fluorescence intensity changes.

Encouraged by the above results, we investigated the tumor‐targeting ability of **T‐BNCy5** in the tumor‐bearing mouse models that were built by subcutaneous injection of cancerous 4T1 cells into BALB/c mice. When the tumor volumes reached up to ≈100 mm^3^, **T‐BNCy5** (100 µm, 100 µL) [note that, even in the concentration, no aggregation could be observed (Figure S14, Supporting Information)] was intravenously injected into the mice via tail vein, and fluorescent signal was monitored at the different time points under a small animal imaging system. For comparison, a synthetic precursor of **T‐BNCy5**, i.e., **A‐BNCy5** [no biotin unit in its molecular structure (Supporting Information)], was also employed in the assay. As shown in **Figure** **5**A, benefiting from its tumor‐targeted biotin unit, **T‐BNCy5** was found to be able to preferentially accumulate in the tumor area only at 15 min post intravenous injection, and the fluorescence signal at the tumor site gradually increased over time and reached up to a maximum at 30 min. After 120 min, the fluorescence at the tumor site almost disappeared. By comparison, no tumor targetability was observed for **A‐BNCy5**, indicative of the important role of the biotin unit in endowing **T‐BNCy5** with the tumor‐targeting ability. Also, we explored the in vivo clearance pathway of **T‐BNCy5**. For this purpose, the tumor‐bearing mice were euthanized at the time points of 0.5, 1, 2, 4, and 6 h, respectively, post intravenous injection of **T‐BNCy5**, and then the tumor and main organs were imaged. As shown in Figure 5B, C, at the time point of 0.5 h, the fluorescence of **T‐BNCy5** was found to mainly accumulate in the tumor, liver, and kidney; at 2 h, the fluorescence in the tumor and liver almost disappeared, but remained obvious in kidney; at 6 h, the fluorescence in kidney nearly disappeared. The result indicates that **T‐BNCy5** could be cleared from the body through renal metabolism. The fluorescence changes in the collected urine also showed a similar trend (Figure 5D,E). The presence of **T‐BNCy5** in urine could be confirmed by the absorption spectra comparison (Figure S15, Supporting Information) and HRMS analysis (Figure S16, Supporting Information). Overall, these results reveal that **T‐BNCy5** could not only specifically target tumors, but also be excreted from the body by the biosafe renal metabolism.^[^ ^7^, ^31^ ^]^

**Figure 6:**
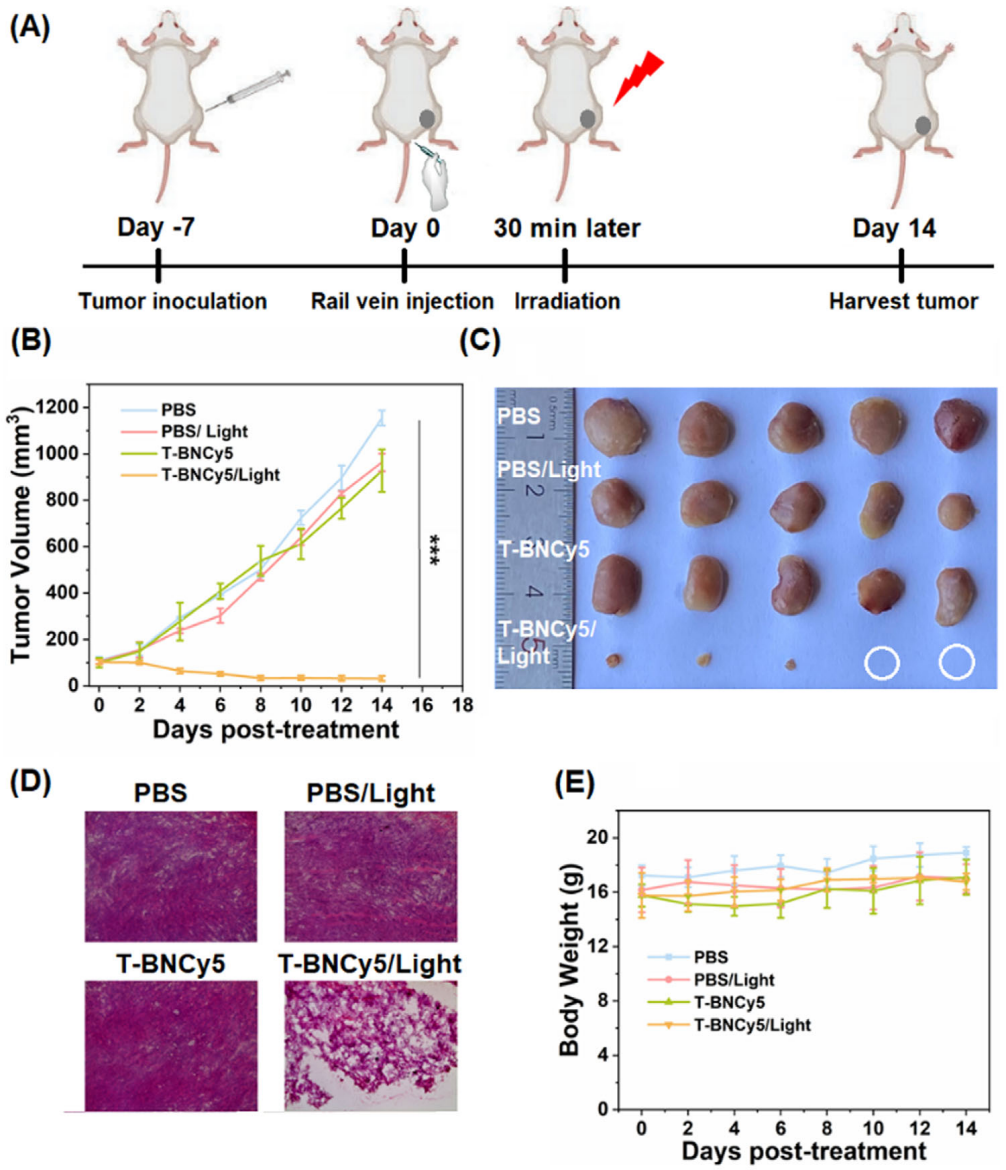
A) Schematic illustration of photodynamic therapy mediated by **T‐BNCy5**. B) Relative tumor volume after different treatments. C) Tumors were harvested from different groups 14 days post‐treatment. D) H&E staining of tumor sections from different treatment groups 14 days post‐treatment. E) Body weights of the mice after different treatments.

Next, we evaluated the antitumor efficacy of **T‐BNCy5** under photoirradiation (660 nm LED light, 100 mW cm^−2^, 20 min) using the 4T1 tumor‐bearing BALB/c mice (**Figure** **6**A). PDT treatment was performed at 30 min post intravenous injection of **T‐BNCy5** (100 µm, 100 µL) when fluorescence in the tumor area was the strongest. In the assays, the 4T1‐tumor‐bearing mice were randomly divided into four groups (n = 5, each group), i.e., PBS group, PBS/light group, **T‐BNCy5** group, and **T‐BNCy5**/light group, respectively. As shown in Figure 6B, C, the tumors in the **T‐BNCy5** group showed remarkable tumor growth (more than 9‐fold after 14 days), and similar cases were also observed in PBS and PBS/light groups. By comparison, tumors in the **T‐BNCy5**/light group almost disappeared, indicating that **T‐BNCy5** possesses excellent PDT efficacy in abating tumors. Further, we stained the frozen sections of tumor tissues from the four groups with hematoxylin and eosin (H&E), which revealed prominent cell death in the **T‐BNCy5**/light group, while no extensive cell damage was observed in other groups (Figure 6D). Moreover, during the PDT process, the mice in the four groups showed slightly increased weight, indicative of the good biocompatibility of **T‐BNCy5** (Figure 6E). The conclusion could further be supported by a series of biosafety assays, including H&E staining of major organs (Figure S17, Supporting Information), hemolysis experiment (Figure S18, Supporting Information), and blood routine and biochemical tests (Figure S19, Supporting Information). Finally, we performed the pharmacokinetic assay post intravenous injection of **T‐BNCy5** in the 4T1 tumor‐bearing mice, which revealed a blood half‐life of 9 min, indicating that the PS could quickly be cleared from blood (Figure S20, Supporting Information).

## 3 Conclusion

In summary, we herein presented a new Type‐I PS, i.e., **T‐BNCy5**, by installing a biotin‐conjugated naphthalimide moiety to the *meso*‐position of an *N*‐benzyl‐functionalized Cy5 dye. Such molecular design endowed **T‐BNCy5** with near‐perfect D‐A orthogonality and strong structural rigidity due to the presence of an intramolecular sandwich‐like π–π interaction, which effectively promotes the SOCT‐ISC to populate an ultralong‐lifetime T_1_ state, thereby improving the O_2_^•−^‐generating ability. In vitro assays revealed that **T‐BNCy5** specifically accumulated in mitochondria, where it not only generated O_2_^•−^ under photoirradiation but also triggered the burst of the most cytotoxic HO^•^ by a cascade of biochemical reactions, which severely damaged mitochondria and induced cell ferroptosis. Whether under normoxia or hypoxia, **T‐BNCy5** displayed a strong cancer cell‐killing ability under photoirradiation with IC_50_ values up to submicromolar levels. Thanks to its biotin unit, **T‐BNCy5** preferentially accumulated at the tumor site, and after a single PDT treatment, it not only demonstrated the potential in ablating tumors but also could be cleared out of the body via biosafe renal metabolism. Overall, **T‐BNCy5** enjoys many merits as a Type‐I PS, including long excitation/emission wavelengths, strong photon‐capturing ability, exclusive O_2_^•−^ generation, triggering cell ferroptosis, excellent tumor‐targeting properties, and bio‐safe renal metabolism, thus being very promising for application in future clinical practice.

## Conflict of Interest

The authors declare no conflict of interest.

## Supporting information



Supporting Information

## Data Availability

The data that support the findings of this study are available in the supplementary material of this article.

## References

[1] a) M. Ethirajan , Y. Chen , P. Joshi , R. K. Pandey , Chem. Soc. Rev. 2011, 40, 340; 20694259 10.1039/b915149b

[2] a) C. Mu , W. Wang , J. Wang , C. Gong , D. Zhang , X. Zhang , Angew. Chem., Int. Ed. 2020, 59, 21515; 10.1002/anie.202010294 32790010

[3] M. Li , K. H. Gebremedhin , D. Ma , Z. Pu , T. Xiong , Y. Xu , J. S. Kim , X. Peng , J. Am. Chem. Soc. 2021, 144, 163. 34963281 10.1021/jacs.1c07372

[4] M. Li , J. Xia , R. Tian , J. Wang , J. Fan , J. Du , S. Long , X. Song , J. W. Foley , X. Peng , J. Am. Chem. Soc. 2018, 140, 14851. 30362735 10.1021/jacs.8b08658

[5] a) D. Chen , Q. Xu , W. Wang , J. Shao , W. Huang , X. Dong , Small 2021, 17, 2006742; 10.1002/smll.202006742 34038611

[6] a) K. Chen , W. Yang , Z. Wang , A. Iagatti , L. Bussotti , P. Foggi , W. Ji , J. Zhao , M. Di Donato , J. Phys. Chem. A 2017, 121, 7550; 28866887 10.1021/acs.jpca.7b07623

[7] D. Zhang , K. X. Teng , L. Zhao , L. Y. Niu , Q. Z. Yang , Adv. Mater. 2023, 35, 202209789. 10.1002/adma.202209789 36861334

[8] Y. Hou , Q. Liu , J. Zhao , Chem. Commun. 2020, 56, 1721. 10.1039/c9cc09058d 31942594

[9] a) J. L. Bricks , A. D. Kachkovskii , Y. L. Slominskii , A. O. Gerasov , S. V. Popov , Dyes Pigm. 2015, 121, 238;

[10] V.‐N. Nguyen , S. Qi , S. Kim , N. Kwon , G. Kim , Y. Yim , S. Park , J. Yoon , J. Am. Chem. Soc. 2019, 141, 16243. 31577431 10.1021/jacs.9b09220

[11] a) J. An , S. Tang , G. Hong , W. Chen , M. Chen , J. Song , Z. Li , X. Peng , F. Song , W.‐H. Zheng , Nat. Commun. 2022, 13, 2225; 35469028 10.1038/s41467-022-29862-9 PMC9038921

[12] M. Lv , X. Lu , Y. Jiang , M. E. Sandoval‐Salinas , D. Casanova , H. Sun , Z. Sun , J. Xu , Y. Yang , J. Chen , Angew. Chem., Int. Ed. 2021, 61, e202113190. 10.1002/anie.202113190 34791747

[13] a) W. Chen , Y. Zhang , H. B. Yi , F. Wang , X. Chu , J. H. Jiang , Angew. Chem., Int. Ed. 2023, 62, e202300162; 10.1002/anie.202300162 36856160

[14] H. Sunahara , Y. Urano , H. Kojima , T. Nagano , J. Am. Chem. Soc. 2007, 129, 5597. 17425310 10.1021/ja068551y

[15] L. Zhao , X. Ren , X. Yan , CCS Chem. 2021, 3, 678.

[16] X. Luo , J. Li , J. Zhao , L. Gu , X. Qian , Y. Yang , Chin. Chem. Lett. 2019, 30, 839.

[17] X. Peng , Z. Yang , J. Wang , J. Fan , Y. He , F. Song , B. Wang , S. Sun , J. Qu , J. Qi , M. Yan , J. Am. Chem. Soc. 2011, 133, 6626. 21476543 10.1021/ja1104014

[18] W. Chi , J. Chen , W. Liu , C. Wang , Q. Qi , Q. Qiao , T. M. Tan , K. Xiong , X. Liu , K. Kang , Y.‐T. Chang , Z. Xu , X. Liu , J. Am. Chem. Soc. 2020, 142, 6777. 32182060 10.1021/jacs.0c01473

[19] X. Xiao , I. Kurganskii , P. Maity , J. Zhao , X. Jiang , O. F. Mohammed , M. Fedin , Chem. Sci. 2022, 13, 13426. 36507154 10.1039/d2sc04258d PMC9682887

[20] a) X. Chen , J. Pang , M. Imran , X. Li , J. Zhao , M. Li , Photochem. Photobiol. Sci. 2021, 20, 69; 33721237 10.1007/s43630-020-00002-w

[21] J. Zhuang , B. Wang , H. Chen , K. Zhang , N. Li , N. Zhao , B. Z. Tang , ACS Nano 2023, 17, 9110. 37144959 10.1021/acsnano.2c12319

[22] a) Z. Liu , H. Zou , Z. Zhao , P. Zhang , G.‐G. Shan , R. T. K. Kwok , J. W. Y. Lam , L. Zheng , B. Z. Tang , ACS Nano 2019, 13, 11283; 31525947 10.1021/acsnano.9b04430

[23] K.‐X. Teng , L.‐Y. Niu , N. Xie , Q.‐Z. Yang , Nat. Commun. 2022, 13, 6179. 36261451 10.1038/s41467-022-33924-3 PMC9582220

[24] W. Chen , Z. Wang , M. Tian , G. Hong , Y. Wu , M. Sui , M. Chen , J. An , F. Song , X. Peng , J. Am. Chem. Soc. 2023, 145, 8130. 37001012 10.1021/jacs.3c01042

[25] I. Noh , D. Lee , H. Kim , C. U. Jeong , Y. Lee , J. O. Ahn , H. Hyun , J. H. Park , Y. C. Kim , Adv. Sci. 2017, 5, 1700481. 10.1002/advs.201700481 PMC5867131 29593951

[26] X. Guo , N. Yang , W. Ji , H. Zhang , X. Dong , Z. Zhou , L. Li , H. M. Shen , S. Q. Yao , W. Huang , Adv. Mater. 2021, 33, 2007778. 10.1002/adma.202007778 34510563

[27] K. X. Teng , D. Zhang , B. K. Liu , Z. F. Liu , L. Y. Niu , Q. Z. Yang , Angew. Chem., Int. Ed. 2024, 63, e202318783. 10.1002/anie.202318783 38258371

[28] M. Zhao , Y. Zhang , J. Miao , H. Zhou , Y. Jiang , Y. Zhang , M. Miao , W. Chen , W. Xing , Q. Li , Q. Miao , Adv. Mater. 2023, 36, 2305243. 10.1002/adma.202305243 37643544

[29] H. Yuan , Z. Han , Y. Chen , F. Qi , H. Fang , Z. Guo , S. Zhang , W. He , Angew. Chem., Int. Ed. 2021, 60, 8174. 10.1002/anie.202014959 33656228

[30] Q. Ren , H. Wang , D. Li , A. Dao , J. Luo , D. Wang , P. Zhang , H. Huang , Adv. Healthcare Mater. 2024, 13, 2304067. 10.1002/adhm.202304067 38597369

[31] Y. Y. Zhao , X. Zhang , Y. Xu , Z. Chen , B. Hwang , H. Kim , H. Liu , X. Li , J. Yoon , Angew. Chem., Int. Ed. 2024, 63, e202411514. 10.1002/anie.202411514 38940633

