# Supplementary material for: Improved Orthogonality in Naphthalimide/Cyanine Dyad Boosts Superoxide Generation: a Tumor‐Targeted Type‐I Photosensitizer for Photodynamic Therapy of Tumor by Inducing Ferroptosis
Source: Adv Sci (Weinh). 2025 Mar 6;12(17):2417179. doi: 10.1002/advs.202417179 (PMC12061322; doi:10.1002/advs.202417179)
Supplement: Supplementary file 1 — Supporting Information [file ADVS-12-2417179-s001.docx]

**Supporting Information**

**Improved Orthogonality in Naphthalimide/Cyanine Dyad Boosts Superoxide Generation: A Tumor-Targeted Type-I Photosensitizer for Photodynamic Therapy of Tumor by Inducing Ferroptosis**

Guangxiao Yao**,^#^** Junfeng Miao,**^#^*** Yingying Huo, and Wei Guo*

*School of Chemistry and Chemical Engineering, Shanxi University, Taiyuan 030006, China.*

*E-mail:* [*miaojunf@sxu.edu.cn*](mailto:miaojunf@sxu.edu.cn) [*guow@sxu.edu.cn*](mailto:guow@sxu.edu.cn)

**1. General information and methods**

All reagents and solvents were purchased from commercial sources and were of the highest grade. Solvents were dried according to standard procedures. All reactions were magnetically stirred and monitored by thin-layer chromatography (TLC). Flash chromatography (FC) was performed using silica gel 60 (200–300 mesh). Absorption spectra were taken on SHIMADZU UV-2600i. Fluorescence spectra were taken on Hitachi F-7000 fluorescence spectrometer. The ^1^H NMR and ^13^C NMR spectra were taken on a Bruker spectrometer, and recorded at 600 and 150 MHz, respectively. The following abbreviations were used to explain the multiplicities: s = singlet; d = doublet; t = triplet; q = quartet; m = multiplet; br = broad. High resolution mass spectra were obtained on a Varian QFT-ESI mass spectrometer. The imaging assays of cells were performed in Zeiss LSM 880+Airyscan Laser Scanning Confocal Microscope. Small animals’ fluorescence imaging was carried out using Bruker’s In-Vivo Fx Pro living imaging system. The nanosecond transient absorption spectra were measured on LP920 laser flash photolysis spectrometer (Edinburgh Instruments Ltd, UK). The signal was digitized with a Tektronix TDS 3012B oscilloscope. All samples were purged with N_2_ for 15 min before the measurements and excited with an Opolette 355II+UV nanosecond pulse laser. The data were processed with the LP900 software. Electron Paramagnetic Resonance (EPR) using Bruker EMX PLUS from Germany. Ethical approval for the animal experiments reported here was obtained from the Animal Ethics Committee of Shanxi University (number: SXULL2024095). The stock solution of all compounds (2 mM) in this work was prepared in DMSO, which was diluted into the corresponding concentrations by water or PBS for different assays.

**2. Synthesis**


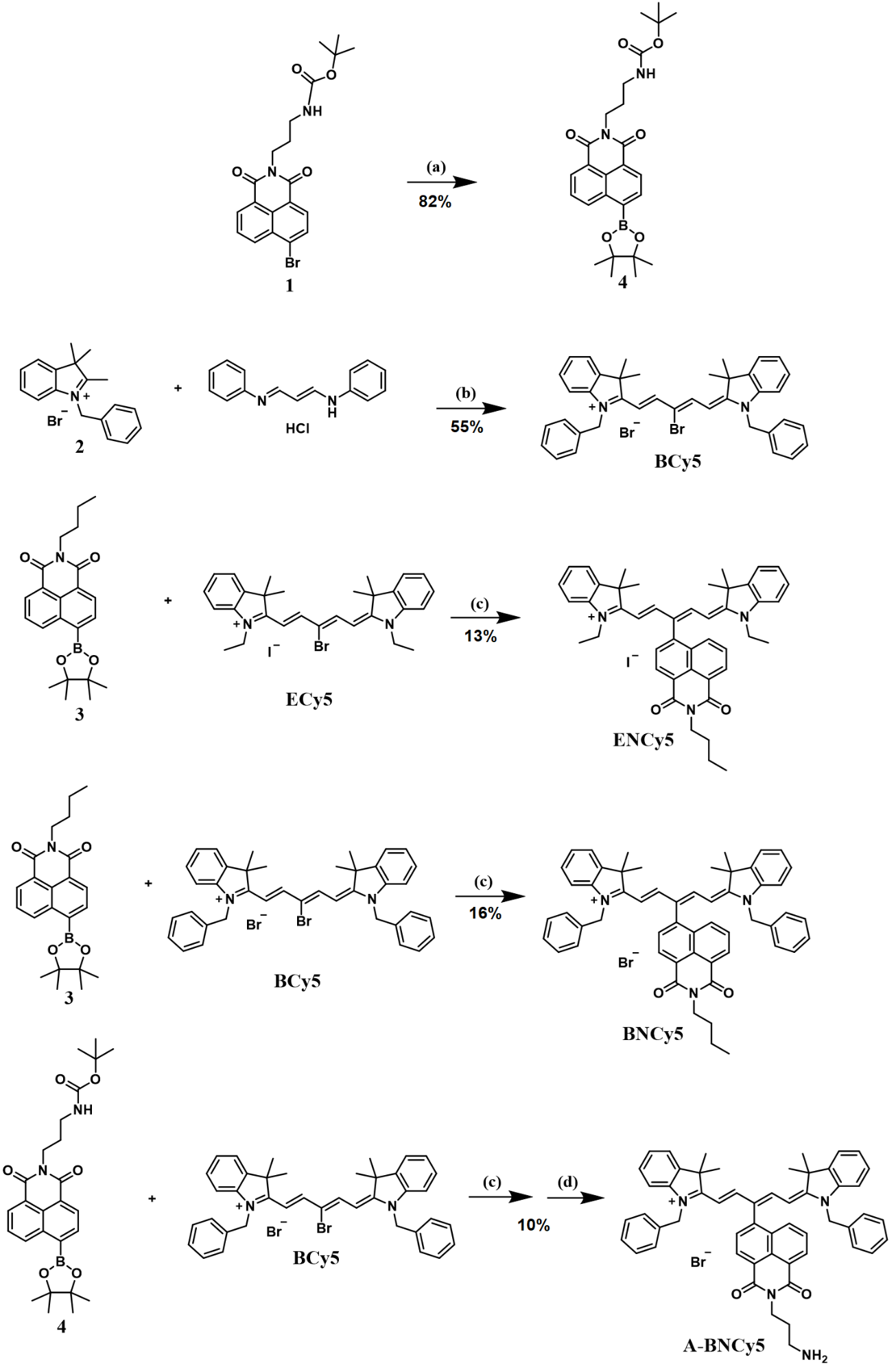


**
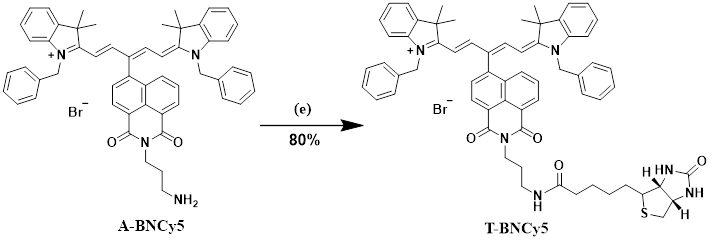
**

**Scheme S1.** Synthetic routes of **BCy5**, **ENCy5**, **BNCy5**, **A-BNCy5**, and **T-BNCy5**. Reagents and conditions: (a) Bis(pinacolato)diborane, Pd(dppf)_2_Cl_2_, KOAc, 1,4-dioxane, 85 °C; (b) NaOAc, Ethanol, 80°C. (c) Pd(PPh_3_)_4_/K_2_CO_3_, Tolune: Ethanol: Water =2: 2: 1, reflux; (d) TFA, CH_2_Cl_2_; (e) Biotin/ EDCl/ HOBt/ DMAP, DMF.

*2.1 Synthesis of Compound* ***4***

**1** (500 mg, 1.15 mmol), Bis(pinacolato)diborane (838 mg, 3.3 mmol), Pd(dppf)_2_Cl_2_ (37 mg, 0.05 mmol) and KOAc (339 mg, 3.45 mmol), were dissolved in 30 mL degassed 1,4-dioxane and stirred at 85℃ for 5 h under an N_2_ atmosphere. After cooling down, the mixture was poured into water, and then extracted with CH_2_Cl_2_*.* The Crude product is concentrated under vacuum, then purified by silica gel column chromatography (PE: EtOAc = 5:1) to give **4** as a light yellow solid (Yield 82%). ^1^H NMR (600 MHz, CDCl_3_) δ 9.13 (d, *J* = 7.4 Hz, 1H), 8.58 (dd, *J* = 21.2, 6.7 Hz, 2H), 8.30 (d, *J* = 7.2 Hz, 1H), 7.81 – 7.75 (m, 1H), 5.26 (s, 1H), 4.29 – 4.23 (m, 2H), 3.20 – 3.12 (m, 2H), 1.96 – 1.90 (m, 2H), 1.45 (s, 12H), 1.29 – 1.20 (m, 9H); ^13^C NMR (151 MHz, CDCl_3_) δ 164.66, 156.05, 135.79, 135.28, 135.20, 131.07, 129.96, 127.89, 127.14, 124.46, 122.36, 84.63, 83.17, 75.04, 37.63, 28.46, 24.99, 24.57.ESI-MS: m/z calcd for [M+H]: 481.2510, Found: 481.2499.

*2.2 Synthesis of Compound* ***BCy5***

A solution of compound **2** (500 mg, 1.5 mmol) and malonaldehyde dianilide hydrochloride (226 mg, 0.75 mmol), NaOAc (308 mg, 3.75 mmol) in 30 mL ethanol was refluxed under N_2_ for 3 h. The reaction mixture was filtered and the filtrate was evaporated under reduced pressure. The residue was purified by silica gel flash chromatography (CH_2_Cl_2_: CH_3_OH = 20:1) to give **BCy5** as a deep blue solid (Yield 55 %). ^1^H NMR (600 MHz, MeOD) δ 8.35 (d, *J* = 13.2 Hz, 2H), 7.59 (d, *J* = 7.4 Hz, 2H), 7.43 – 7.29 (m, 16H), 6.47 (d, *J* = 13.2 Hz, 2H), 5.41 (s, 4H), 1.78 (s, 12H); ^13^C NMR (151 MHz, MeOD) δ 176.87, 151.66, 143.81, 142.86, 135.63, 130.34, 130.02, 129.44, 127.90, 127.24, 123.74, 117.82, 112.81, 104.82, 51.17, 27.60. ESI-MS: m/z calcd for [M]^+^: 613.2213, Found: 613.2186.

2.3 Synthesis of Compound **ENCy5**

**ECy5** (250 mg, 0.40 mmol), compound **3** (152 mg, 0.40 mmol), K_2_CO_3_ (359 mg, 2.6 mmol), and Pd(PPh_3_)_4_ (231 mg, 0.2 mmol) were dissolved in 7.5 mL degassed toluene: ethanol: water 2: 2: 1 mixed solvent and the mixture was refluxed for 2 hours under an N_2_ atmosphere. TLC point plates were monitored and the reaction was completely cooled to room temperature. The organic solution was washed with brine, dried over Na_2_SO_4_, and evaporated. The residue was purified by silica gel flash chromatography (CH_2_Cl_2_: CH_3_OH = 20:1) to give **ENCy5** as a deep blue solid (Yield 13 %).^1^H NMR (600 MHz, MeOD) δ 8.77 (d, *J* = 6.5 Hz, 1H), 8.65 (dd, *J* = 28.4, 10.1 Hz, 3H), 8.21 (d, *J* = 6.9 Hz, 1H), 7.83 (s, 2H), 7.53 (d, *J* = 6.7 Hz, 2H), 7.38 (d, *J* = 6.4 Hz, 2H), 7.29 (d, *J* = 6.9 Hz, 2H), 7.21 (d, *J* = 7.1 Hz, 2H), 5.40 (dd, *J* = 12.5, 6.0 Hz, 2H), 4.24 – 4.18 (m, 2H), 3.63 (s, 4H), 1.83 (d, *J* = 10.6 Hz, 12H), 1.51 – 1.46 (m, 2H), 1.30 (dd, *J* = 24.4, 9.8 Hz, 2H), 1.06 – 1.00 (m, 3H), 0.89 (s, 6H);^13^C NMR (151 MHz, MeOD) δ 173.56, 163.98, 153.29, 141.47, 141.38, 131.81, 131.20, 130.86, 130.05, 129.77, 128.72, 128.42, 127.43, 125.39, 123.34, 122.73, 122.13, 110.63, 100.64, 49.45, 39.81, 38.43, 29.90, 26.27, 26.21, 20.01, 12.81, 10.56.ESI-MS: m/z calcd for [M]^+^: 622.3742, Found: 622.3744.

*2.4 Synthesis of Compound* ***BNCy5***

**BCy5** (250 mg, 0.36 mmol), compound 3 (137 mg, 0.36 mmol), K_2_CO_3_ (323 mg, 2.34 mmol), and Pd(PPh_3_)_4_ (208 mg, 0.18 mmol) were dissolved in 6.25 mL degassed toluene: ethanol: water 2: 2: 1 mixed solvent and the mixture was refluxed for 2 hours under an N_2_ atmosphere. TLC point plates were monitored and the reaction was completely cooled to room temperature. The organic solution was washed with brine, dried over Na_2_SO_4_, and evaporated. The residue was purified by silica gel flash chromatography (CH_2_Cl_2_: CH_3_OH = 20:1) to give **BNCy5** as a deep blue solid (Yield 16 %). ^1^H NMR (600 MHz, MeOD) δ 8.54 (dd, *J* = 7.2, 6.8 Hz, 4H), 7.76 (d, *J* = 8.3 Hz, 1H), 7.55 (dd, *J* = 15.4, 7.8 Hz, 3H), 7.41 – 7.34 (m, 1H), 7.33 – 7.27 (m, 1H), 7.03 (t, *J* = 6.9 Hz, 1H), 6.90 (t, *J* = 7.2 Hz, 1H), 6.51 (d, *J* = 7.2 Hz, 1H), 5.21 (d, *J* = 14.3 Hz, 1H), 4.81 (s, 1H), 4.78 (s, 1H), 4.33 – 4.29 (m, 1H), 1.82 (s, 3H), 1.55 (dd, *J* = 13.6, 6.4 Hz, 1H), 1.38 – 1.32 (m, 1H), 1.07 (t, *J* = 7.2 Hz, 1H)；^13^C NMR (151 MHz, MeOD) δ 175.72, 165.58, 165.50, 154.55, 144.05, 142.66, 135.39, 132.92, 132.76, 132.44, 130.28, 129.86, 129.81, 129.23, 128.86, 128.81, 127.57, 126.86, 123.73, 123.56, 112.18, 104.41, 50.78, 49.62, 41.22, 30.79, 27.92, 27.74, 21.48, 14.28.ESI-MS: m/z calcd for [M]^+^: 786.4055, Found: 786.4023.

*2.5* *Synthesis of Compound* ***A-BNCy5***

**BCy5** (250 mg, 0.36 mmol), compound **4** (173 mg, 0.36 mmol), K_2_CO_3_ (323 mg, 2.34 mmol) and Pd(PPh_3_)_4_ (208 mg, 0.18 mmol) were dissolved in 6.25 mL degassed toluene: ethanol: water 2: 2: 1 mixed solvent and the mixture was refluxed for 2 hours under an N_2_ atmosphere. TLC point plates were monitored and the reaction was completely cooled to room temperature. The organic solution was washed with brine, dried over Na_2_SO_4_, and evaporated. The residue was purified by silica gel flash chromatography (CH_2_Cl_2_: CH_3_OH = 20:1). Then the compound was taken up in 1 mL of CH_2_Cl_2_ and 1 mL of TFA was added. The mixture was stirred for 2 h at room temperature. The residue was purified by silica gel chromatography (CH_2_Cl_2_: CH_3_OH = 10:1) to give **A-BNCy5** as a deep blue solid (Yield 10 %). ^1^H NMR (600 MHz, MeOD) δ 8.57 (d, *J* = 12.6 Hz, 4H), 7.83 (d, *J* = 7.6 Hz, 1H), 7.58 (dd, *J* = 15.5, 7.5 Hz, 3H), 7.37 (t, *J* = 6.2 Hz, 3H), 7.29 (dd, *J* = 22.5, 7.1 Hz, 4H), 7.05 (dd, *J* = 5.4, 1.0 Hz, 2H), 6.94 (s, 4H), 6.55 (d, *J* = 5.5 Hz, 4H), 5.22 (d, *J* = 13.9 Hz, 2H), 4.79 (s, 4H), 4.46 – 4.38 (m, 2H), 3.18 – 3.10 (m, 2H), 2.24 (d, *J* = 4.5 Hz, 2H), 1.82 (s, 12H), 1.36 – 1.24 (m, 2H);^13^C NMR (151 MHz, MeOD) δ 174.29, 164.40, 164.28, 153.22, 142.50, 141.20, 134.00, 131.71, 131.24, 130.91, 129.69, 129.24, 128.54, 128.40, 127.47, 127.44, 125.54, 122.71, 122.29, 110.77, 102.75, 49.37, 46.99, 37.35, 36.87, 26.42, 26.26, 26.20.ESI-MS: m/z calcd for [M]^+^: 787.4007, Found: 787.4000.

*2.6. Synthesis of Compound* ***T-BNCy5***

**A-BNCy5** (30 mg, 0.035 mmol), Biotin (17 mg, 0.07 mmol), EDCl (8.5 mg, 0.044 mmol), HOBt (7.1 mg, 0.051 mmol), and DMAP (5.4 mg, 0.044 mmol) were added to 2 mL dry DMF and the mixture was stirred at room temperature for 2 hours under an N_2_ atmosphere. After cooling down, the mixture was poured into water, and then extracted with CH_2_Cl_2_*.* The residue was purified by silica gel chromatography (CH_2_Cl_2_: CH_3_OH = 10:1) as eluent to give **T-BNCy5** (Yield 80 %). ^1^H NMR (600 MHz, MeOD) δ 8.57 – 8.52 (m, 4H), 8.15 – 8.10 (m, 1H), 7.77 (d, *J* = 8.5 Hz, 1H), 7.56 (d, *J* = 7.3 Hz, 3H), 7.40 – 7.26 (m, 8H), 7.04 (t, *J* = 7.2 Hz, 2H), 6.92 (t, *J* = 7.4 Hz, 4H), 6.53 (d, *J* = 7.2 Hz, 4H), 5.21 (d, *J* = 14.2 Hz, 2H), 4.80 (d, *J* = 13.2 Hz, 4H), 4.49 – 4.45 (m, 1H), 4.36 – 4.30 (m, 3H), 4.10 (dd, *J* = 14.2, 7.1 Hz, 1H), 3.41 – 3.37 (m, 2H), 3.26 – 3.21 (m, 1H), 2.91 (dd, *J* = 12.7, 4.9 Hz, 1H), 2.68 (d, *J* = 12.7 Hz, 1H), 2.29 (t, *J* = 7.1 Hz, 2H), 2.09 – 2.04 (m, 2H), 1.82 (s, 12H), 1.74 – 1.70 (m, 2H), 1.64 (dd, *J* = 13.4, 7.0 Hz, 2H), 1.51 (dt, *J* = 13.6, 6.9 Hz, 2H);^13^C NMR (151 MHz, MeOD) δ 176.21, 175.67, 166.14, 165.61, 165.54, 154.57, 144.03, 142.64, 135.39, 133.01, 132.84, 132.57, 130.01, 129.84, 128.89, 126.95, 126.89, 124.36, 123.73, 123.72, 123.55, 112.20, 104.37, 63.31, 61.69, 56.99, 54.85, 50.76, 49.89, 41.13, 38.28, 36.97, 29.73, 29.53, 29.41, 27.88, 27.71, 26.95.ESI-MS: m/z calcd for [M]^+^: 1013.4783, Found: 1013.4786.

**3. Fluorescence quantum yield determination**

Fluorescence quantum yields were determined with Cy5 (Φ_f_ = 0.15 in MeOH) was used as a reference. The quantum yield was calculated using the following equation.

$$\Phi_{\Delta}^{\alpha}=\Phi_{\Delta}^{ref}\times\frac{S_{a}}{S_{ref}}\times\frac{A_{ref}}{A_{a}}\times\left( \frac{n_{a}}{n_{ref}} \right)^{2}$$

Where ***A****_a_* and ***A****_ref_* are the absorbance at the reference excitation wavelength, ***S****_a_* and ***S****_ref_* are the corresponding integrated fluorescence intensity, and ***n****_a_* and ***n****_ref_* are the solvent refractive indexes of the sample and reference, respectively. The absorbance of all samples to be tested at their respective excitation wavelengths was required to be below 0.05.

**4. Singlet oxygen (^1^O_2_) detection**

9,10-Anthracenediyl-bis(methylene)dimalonic Acid (ABDA, as a water-soluble ^1^O_2_ scavenger, abs ~ 1.00) and photosensitizers (5 μM) were dissolved in PBS (10 mM, pH = 7.4), which was subjected to continuous light irradiation. The absorption of ABDA at 378 nm was recorded every 10 seconds to obtain the decay rate of the photosensitizing process. The measurements were performed using 660 nm LED light (20 mW cm^−2^) for **ENCy5**, **BNCy5**, and **T-BNCy5**.

**5. Superoxide radical (O_2_^•−^) detection**

To evaluate the O_2_^•−^ generation, dihydrorhodamine 123 (DHR123) was used as the specific indicator,^[1]^ which can be converted to Rhodamine 123 in the presence of O_2_^•−^ and emits strong red fluorescence at 529 nm. The cuvette containing a water solution of DHR123 (10 μM) and **ENCy5** or **BNCy5** or **T-BNCy5** (5 μM) was exposed to 660 nm LED light illumination (20 mW cm^−2^) for 10 seconds, and the emission spectra were observed immediately after irradiation (λ_ex_ = 495 nm).

**6. Hydroxy radical (HO^•^) detection**

To evaluate the HO^•^ generation, Hydroxyphenyl fluorescein (HPF) was used as the specific indicator,^[2]^ which can be converted to Hydroxyphenyl fluorescein in the presence of HO^•^ and emits strong green fluorescence at 520 nm. The cuvette containing a water solution of HPF (10 μM) and **ENCy5** or **BNCy5** or **T-BNCy5** (10 μM) and was exposed to 660 nm LED light illumination (20 mW cm^-2^) for 10 seconds, and the emission spectra were observed immediately after irradiation (λ_ex_ = 485 nm).

**7. Absorption spectra change of the water solution of NADH in the presence of T-BNCy5 under photoirradiation**

The cuvette containing a mixed water solution of NADH (180 μM) and **T-BNCy5** (10 μM) was exposed to 660 nm LED light illumination (20 mW cm^-2^) for 120 seconds, and the absorption spectra were recorded every 20 seconds.

**8. DFT Calculation**

All calculations were performed at the m062x/6-31g* level with Gaussian 09 with an applied polarizable continuum model of solvation of Water. Ground-state geometry and frontier molecular orbitals of **T-BNCy5** were calculated by using density functional theory (DFT). Geometry optimizations and energy levels of the excited states were calculated by using time-dependent DFT (TDDFT). The triplet state was calculated by setting the spin as a triplet.

**9. EPR measurement of Superoxide radical (O_2_^•−^)**

Electron Spin Resonance (ESR) spectroscopy is a sensitive and non-invasive technique widely used to detect the generation of the superoxide anion (O_2_^•-^). Spin traps, such as DMPO (5,5-dimethyl-1-pyrroline N-oxide), react with O_2_^•-^ to form stable radical adducts that produce well-defined ESR signals.^[3]^ ESR spectroscopy was used to detect the electron spin resonance signals from four different sets of samples. a) 100 μM of KO_2_ was dispersed in DMSO containing 100 mM of DMPO and illuminated with a xenon lamp (300 W); b) 100 μM of **T-BNCy5** was dispersed in water containing 100 mM of DMPO and illuminated with a xenon lamp (300 W); c) 100 μM of **T-BNCy5** was dispersed in water containing 100 mM of DMPO without light-irradiation; d) 100 mM of DMPO was dissolved in water and illuminated with a xenon lamp (300 W).

**10. EPR measurement of singlet oxygen (^1^O_2_)**

Electron Spin Resonance (ESR) spectroscopy is a sensitive and non-invasive technique widely used to detect the generation of singlet oxygen (^1^O_2_).^[4]^ Spin traps, such as TEMP (2,2,6,6-Tetramethyl-4-piperidone hydrochloride), react with ^1^O_2_ to form stable radical adducts that produce well-defined ESR signals. ESR spectroscopy was used to detect the electron spin resonance signals from four different sets of samples. a) 1 mM of methylene blue (MB) was dispersed in water containing 100 mM of TEMP and illuminated with a xenon lamp (300W); b) 1 mM of **T-BNCy5** was dispersed in water containing 100 mM of TEMP and illuminated with a xenon lamp (300W); c) 1 mM of **T-BNCy5** was dispersed in water containing 100 mM of TEMP without light-irradiation; d) 100 mM of TEMP was dissolved in water and illuminated with a xenon lamp (300W).

**11. Cyclic voltammetry measurement**

A cyclic voltammetry experiment was performed using a three-electrode setup.^[5]^ The working electrode consisted of a platinum-carbon composite. The auxiliary electrode was a platinum wire, while an Ag/AgNO_3_ electrode served as the reference electrode. The electrolyte used was
acetonitrile with 0.10 M Bu_4_N[PF_6_], and the scan rate was set at 50 mV/s. Ferrocene (Fc/Fc^+^) was employed as an external reference standard.

**12. Electronchemical study**

$${\Delta G}_{S}=-\frac{e^{2}}{4\pi\varepsilon_{s}\varepsilon_{0}R_{CC}}-\frac{e^{2}}{{8\pi\varepsilon}_{0}}\left( \frac{1}{R_{D}}+\frac{1}{R_{A}} \right)\left( \frac{1}{\varepsilon_{REF}}-\frac{1}{\varepsilon_{S}} \right)$$

$$\Delta G_{\mathrm{PeT}}=e\left[ E_{OX}-E_{RED} \right]-E_{00}+\Delta G_{S}$$

Where ${\Delta G}_{S}$ is the static Coulombic energy, $\Delta G_{\mathrm{PeT}}$ is the Gibbs free-energy change of charge separation process. *e* represents the electron charge, $E_{OX}$ is the half-wave potential for one-electron reduction of the electron donor unit, $E_{RED}$ is the half-wave potential for one-electron reduction of the electron acceptor unit, $E_{00}$ is the energy approximated with the crossing point of the normalized UV-vis absorption spectra and fluorescence emission spectra, ε_S_ is the static dielectric constant of the solvent, ACN (*ε*_S_ = 37.5), water (*ε*_S_ = 78.5). R_CC_ is the center-to-center separation distance between the electron donor and acceptor, determined by the conformation optimized with DFT calculations, R_D_ and R_A_ are the radius of the electron donor and acceptor, respectively, *ε*_REF_ is the static dielectric constant of the solvent, *ε*_0_ is permittivity of vacuum.^[6]^

**13. Cell culture**

MCF-7 cells and COS-7 cells were incubated on the cell culture plate in DMEM medium supplemented with 10% FBS;4T1 cells were incubated on the cells culture plate in RPMI-1640 medium supplemented with 10% FBS, at 37 ℃ in a humidified, 5% CO_2_ atmosphere. The cells were plated on a glass bottom cell culture dish (35 mm) and allowed to adhere for 12 hours. Before experiments, cells were washed with phosphate buffer saline (PBS 10 mM) 3 times.

**14. In vitro phototoxicity studies under normoxia**

MCF-7 cells and COS-7 cells were seeded in 96-well cell culture plates at a density of 5000 cells per well. After 24 h of cell attachment, the plates were washed with PBS (10 mM) and then added with DMEM containing **T-BNCy5** at different concentrations. The cells treated with **T-BNCy5** were cultured for 2 h. The plates were washed with PBS (10 mM), and then added with 100 μL fresh DMEM, followed by 660 nm LED light (for **T-BNCy5**, 20 mW cm^-2^, 20 min). After light irradiation, cells were allowed to continuously grow for 24 h. Then, 10 μL MTT solutions (5 mg/mL) in PBS (10 mM) were added to each well. After incubating the cells for another 4 h, the medium was removed, and DMSO (100 μL) was added to each well and gently shaken for 10 min at room temperature. Finally, the absorbance of 550 nm was measured with a Bio-Rad microplate reader, and the cell viability was calculated by the following equation:

Cell viability (%) = (OD_𝑝𝑠_ − OD_𝑏𝑙𝑎𝑛𝑘 𝑐𝑜𝑛𝑡𝑟𝑜𝑙_/OD_𝑐𝑜𝑛𝑡𝑟𝑜𝑙_ − OD_𝑏𝑙𝑎𝑛𝑘 𝑐𝑜𝑛𝑡𝑟𝑜𝑙_) × 100%

To evaluate the dark toxicity, no light irradiation was applied to this experiment, and all other steps were the same.

**15. In vitro phototoxicity studies under hypoxia**

MCF-7 cells were first seeded in 96-well cell culture plates at a density of about 5000 cells per well. After incubation in a 21% O_2_ atmosphere at 37℃ for 12 h, the cells were further cultured in an incubator chamber (MIC-101, Billups-Rothenberg) at 37℃ in a humidified, 2% O_2_, 5% CO_2_, and 93% N_2_ atmosphere for 12 h. Then, DMEM containing **T-BNCy5** at different concentrations was added into each well, and cultured for 2 hr. The plates were washed with PBS (10 mM), and then added with 100 μL fresh DMEM, followed by continuous irradiation with 660 nm LED (for **T-BNCy5** 20 mW cm^-2^, 20 min). All these treatments were conducted under hypoxia (2% O_2_). After light treatment, cells were allowed to continue growing for 24 hours. Other procedures were the same as those of phototoxicity evaluation under normoxia. To evaluate the dark toxicity, no light irradiation was applied to this experiment, and all other steps were the same.

The effect of various inhibitors of cell death on MCF-7cells was assessed by pre-incubating them with Z-VAD-FMK (50 μM), 3-MA (100 μM), Fer-1 (50 μM), VX-765 (100 μM) and Nec-1 (50 μM) for 4 hours. After this pre-incubation period, the cells were co-incubated with **T-BNCy5** (1 μM). Following the 2h co-incubation period, the cells were exposed to 660 nm laser irradiation (power density = 20 mW cm^-2^, 20 min), and subsequently, the cell viability was determined.

**16. Cell co-staining assay**

To evaluate the subcellular localization of **T-BNCy5**, MCF-7 cells were co-incubated with **T-BNCy5** (1.0 µM)/MitoTracker™ Deep Red FM (50 nM) or **T-BNCy5** (1.0 µM)/LysoTracker® Deep Red (40 nM) in DMEM for 30 min and after washed with PBS 3 times, the cells were imaged under CLSM. For green channel, emission was collected from 493 nm to 550 nm (λ_ex_ = 488 nm), and for red channel, emission was collected from 638 nm to 747 nm (λ_ex_ = 633 nm).

**17. Intracellular singlet oxygen imaging**

SOSG was employed as the intracellular ^1^O_2_ indicator, which can emit green fluorescence in the presence of ^1^O_2_.^[7]^ MCF-7 cells were plated onto 35 mm confocal dishes and incubated with 1 μM **T-BNCy5** in DMEM for 2 h, and then stained with 10 μM SOSG for another 30 min. After washed with PBS (10 mM) three times, the cells were irradiated in DMEM with 660 nm LED light (20 mW cm^-2^, 20 min). The excitation wavelength for SOSG was 488 nm and emission was collected at 493-565 nm.

**18. Intracellular superoxide radical imaging**

DHE was employed as the intracellular O_2_^•−^ indicator, which can emit red fluorescence in the presence of O_2_^•−^.^[8]^ MCF-7 cells were plated onto 35 mm confocal dishes and incubated with 1 μM **T-BNCy5** in DMEM for 2 h, and then stained with 10 μM DHE for another 30 min. After washed with PBS three times, the cells were irradiated in DMEM with 660 nm LED light (20 mW cm^-2^, 20 min). The emission for DHE was collected at 560-660 nm (λ_ex_ = 488 nm).

**19. JC-1 staining assays**

MCF-7 cells were seeded and cultured in 35 mm confocal dishes for 24 h in DMEM 1 μM **T-BNCy5** was then added into the medium and incubated with cells for 2 h. After washed three times with PBS, the cells were irradiated with 660 nm LED light (20 mW cm^−2^, 20 min) in DMEM and then stained by JC-1 kit according to the instruction manual. As a control, a similar assay without light irradiation was performed. Normal cells with a high mitochondrial membrane potential show strong fluorescence in the red channel (aggregates of JC-1), and ferroptotic cells with a low mitochondrial membrane potential show poor fluorescence in the red channel but strong fluorescence in the green channel (monomer of JC-1). For the green channel, emission was collected from 493 nm to 550 nm (λ_ex_ = 488 nm), and for the red channel, emission was collected from 560 nm to 660 nm (λ_ex_ = 488 nm).

**20. Intracellular Hydroxy radical imaging**

HPF was employed as the intracellular HO^•^ indicator, which can emit red fluorescence in the presence of HO^•^.^[2]^ MCF-7 cells were plated onto 35 mm confocal dishes and incubated with 1 μM **T-BNCy5** in DMEM for 2 h, and then stained with 5 μM HPF for another 30 min. After washed with PBS three times, the cells were irradiated in DMEM with 660 nm LED light (20 mW cm^-2^, 20 min). The excitation wavelength for HPF was 488 nm and emission was collected at 493-600 nm.

**21. Live/dead cell co-staining**

Calcein AM/propidium iodide (PI) Double Stain Kit was used to assess the cytotoxicity caused by **PS** under light, in which calcein AM (green fluorescence) and propidium iodide (PI; red fluorescence) were intended to mark live and dead cells,^[7]^ respectively. MCF-7 cells were plated onto 35 mm confocal dishes and incubated with 1 μM **T-BNCy5** for 2 h, and irradiated with or without 660 nm LED light (20 mW/cm^2^) for 20 min, then stained with 5 μM Calcein AM and 1 μM PI. Then, confocal fluorescence imaging was performed. The excitation wavelength for Calcein AM was 488 nm and emission was collected at 500-550 nm, the excitation wavelength for PI was 561 nm and emission was collected at 570-620 nm.

**22. Superoxide dismutase (SOD) inhibition assay**

MCF-7 cells were plated onto 35 mm confocal dishes and incubated with 20 μM 2-methoxyestradiol in DMEM for 2 h and then co-stained with 1 μM **T-BNCy5** for another 2 h.^[9]^ After washed with PBS three times, the cells were irradiated in DMEM with 660 nm LED light (20 mW cm^−2^, 20 min). Finally, DHE and HPF were used to assess the intracellular O_2_^•−^ and OH^•^ levels, respectively. For DHE, from 560 nm to 660 nm (λ_ex_ = 488 nm). For HPF, from 493 nm to 600 nm (λ_ex_ = 488 nm).

**23. Cellular NADH Determination**

Intracellular NADH determination Experiments to determine the NADH changes in MCF-7 cells treated with different concentrations of **T-BNCy5** were performed using the NAD^+^/NADH assay kit. In brief, MCF-7 cells were first seeded in six-well cell plates at a density of about 50000 cells per well at 21% O_2_ atmosphere at 37℃ for 12 h. Then, DMEM containing **T-BNCy5** at different concentrations was added into each well, and cultured for 2 hr. The plates were washed with PBS (10 mM), and then added with 100 μL fresh DMEM, followed by continuous irradiation with 660 nm LED (for **T-BNCy5** 20 mW cm^-2^, 20 min). Following a 4-hour incubation, we used the NAD^+^/NADH assay kit to assess NADH levels. 500µL of NAD^+^/NADH extraction buffer was added to the cells, sonicated for 1min, then boiled for 5min, cooled in an ice bath and centrifuged at 1000g for 10 min at 4 °C, then added to a 96-well plate containing the assay reagent, and then the NADH level was quantified by measuring the absorbance at 570 nm using an enzyme marker.

**24. LPO detection in cells**

Lipid peroxidation in MCF-7 cells was assessed using confocal imaging with C11-BODIPY581/591 staining.^[10]^ Cells were seeded in confocal dishes and allowed to culture overnight. After removing the culture medium, cells were subjected to various treatments: Control (no treatment), **T-BNCy5** (1 μM), **T-BNCy5** + Light, and **T-BNCy5** + Light + Fer-1 (50 μM). Following treatment, cells were washed with PBS and then incubated with C11-BODIPY581/591 (10 μM) in a serum-free medium for 30 minutes in the incubator. Subsequently, confocal microscopy was employed to capture fluorescence images of the treated cells. For the green channel, emission was collected from 505 nm to 550 nm (λ_ex_ = 488 nm), and for the red channel, emission was collected from 580 nm to 640 nm (λ_ex_ = 561 nm).

**25. Immunofluorescence study of GPX4**

MCF-7 cells were seeded in confocal dishes and allowed to culture overnight. After removing the culture medium, cells were subjected to various treatments: Control (no treatment), **T-BNCy5** (1 μM), and **T-BNCy5** (1 μM) + Light. The cells were gently washed with PBS and fixed with 4% paraformaldehyde for 20 min. The cells were washed twice with PBS for 5 mins, followed by treatment with permeation buffer (PBS with 0.5% Triton X-100) for 20 min. The permeation buffer was removed and rinsed with PBS for a couple of times. The samples were blocked using 5% BSA in PBS for 60 min in room temperature. The blocking solution was removed, followed by treatment with primary antibody specific for GPX4 with a desired dilution (1:200) in 4 °C for 16 h. The samples were washed with PBS twice, followed treatment with secondary antibody conjugated to Alexa Fluor-594 for 1.5 h in room temperature. The cells were gently washed, followed by counterstaining with Mounting Medium，antifading (with DAPI) and imaged using confocal microscope.

**26. In vivo imaging and PDT assays of tumor**

Specific female BALB/c nude mice, 4-6 weeks of age, originally purchased from the SPF (Beijing) Biotechnology Co., Ltd, were used to establish a mammary cancer mouse model. Briefly, 4T1 cells were injected subcutaneously into the selected positions to establish the mammary tumor model of BALB/c mice. Tumors were allowed to grow to about 100 mm^3^ in volume before being used for in vivo imaging and photodynamic therapy. The water solution of **T-BNCy5** or **A-BNCy5** (100 μM) for intravenous injection was prepared by adding 50 μL stock solution of **T-BNCy5** or **A-BNCy5** (2 mM in DMSO) into 1 mL deionized water. In this case, **T-BNCy5** still shows good water solubility, as evidenced by the concentration-dependent changes in absorption spectra that match well with the Lambert-Beer law (Figure S14).

For in vivo imaging assay, the 4T1 tumor-bearing BALB/c nude mice were intravenously injected with **T-BNCy5** or **A-BNCy5** (100 μM, 100 μL) by tail vein, and the in vivo fluorescence signals were monitored at different post-injection times (0, 15min, 30min, 45min, 60min, 75min, 90min, 120min) under a small animals imaging system. For in vivo PDT assay, the 4T1 tumor-bearing BALB/c nude mice were divided into four groups: PBS group [(administrated alone with PBS (100 μM, 100 μL]; PBS/Light group [administrated with PBS (100 μM, 100 μL), followed by light irradiation (660 nm LED, 100mW cm^-2^, 20 min)]; **T-BNCy5** group [administrated alone with **T-BNCy5** (100 μM,100 μL)]; **T-BNCy5**/Light group [administrated with **T-BNCy5** (100 μM, 100 μL), followed by light irradiation (660 nm LED, 100mW cm^-2^, 20 min)]. 30 minutes post intravenous injection, light irradiation was performed. During the treatment period, the tumor volume and body weight of all mice were measured every other day. The greatest longitudinal diameter (length) and the greatest transverse diameter (width) were used to calculate the tumor volume. Tumor volume = width × width × length/2. At 14 days post-treatment, tumors were harvested and weighed. For histological analysis, the H&E staining of tumor slices was carried out. Each group contained 5 mice.

**27. Pharmacokinetic studies**

Urine samples were collected in tubes as a control before injection. Mice were intravenously injected with **T-BNCy5** (100μM,100μL), and urine samples were taken at 1, 2, 3, 4, 6, 8, and 12 h after the injection. Urine centrifugation at 3000 r.p.m for 15 min. Quantitative analysis of **T-BNCy5** in the urine was performed using fluorescence imaging.

**28. Biocompatibility studies**

Following a 24-hour injection of **T-BNCy5** and **PBS**, major organs including the heart, liver, spleen, lungs, and kidneys were harvested from mice and cryo-sectioned as 10 μm thicknesses, followed by hematoxylin-eosin (H&E) staining.

**29. Supplementary Spectra**


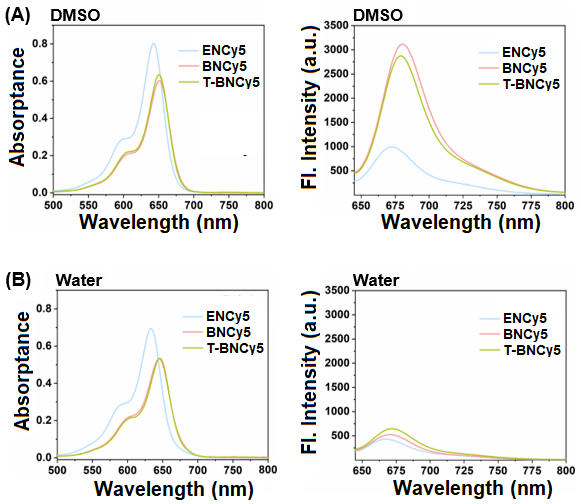


**Figure S1.** The absorption and fluorescence spectra of **ENCy5**, **BNCy5**, and **T-BNCy5** (all 4 μM) in (A) DMSO and (B) water, respectively.


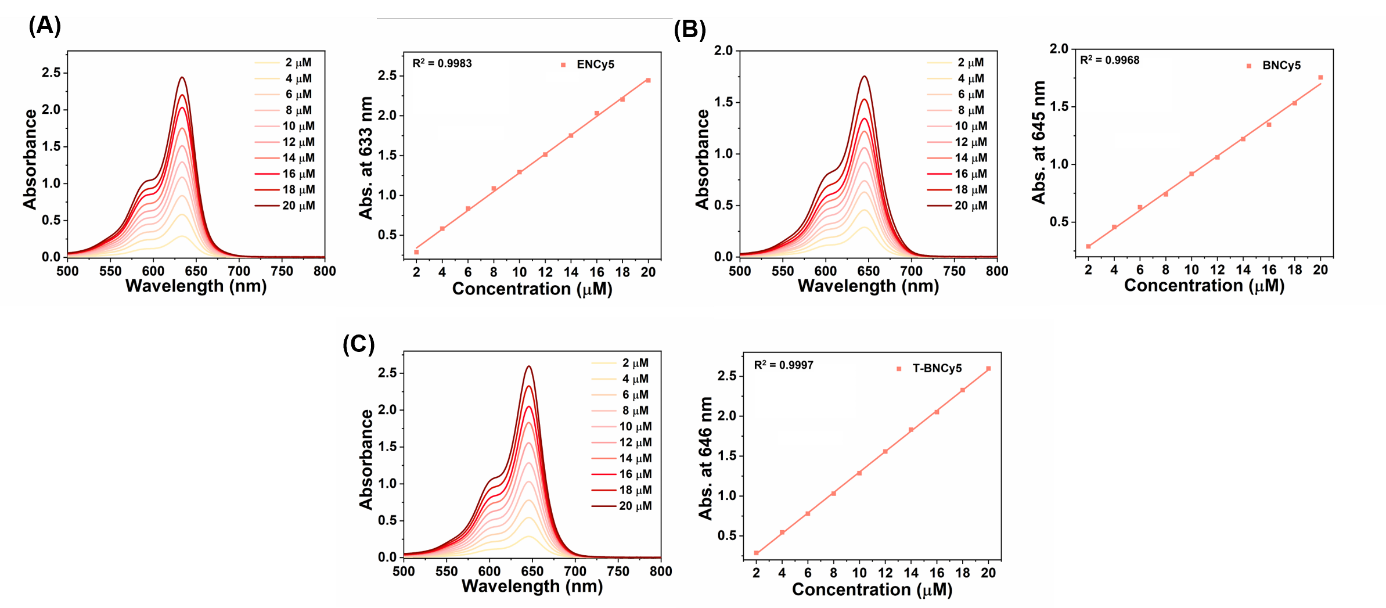


**Figure S2.** Absorption spectra of **ENCy5** (A), **BNCy5** (B), and **T-BNCy5** (C) in aqueous solution with different concentrations (from 2 μM to 20 μM). The aqueous solutions were prepared by adding their stock solutions (2 mM in DMSO) into the deionized water.


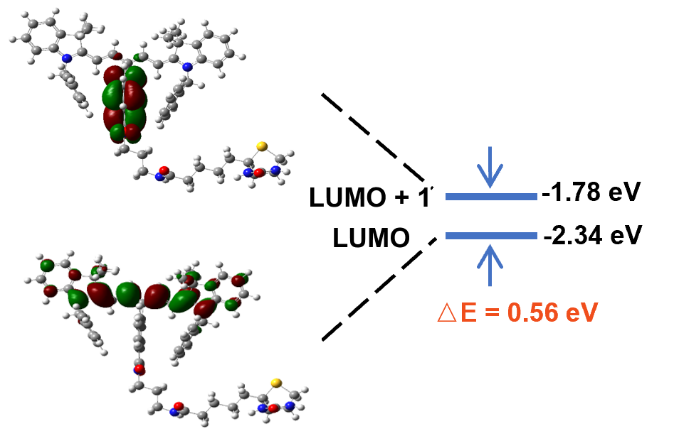


**Figure S3.** The frontier molecular orbitals of **T-BNCy5** are calculated by DFT at the m062x/6-31g* level of Gaussian 09 in water.


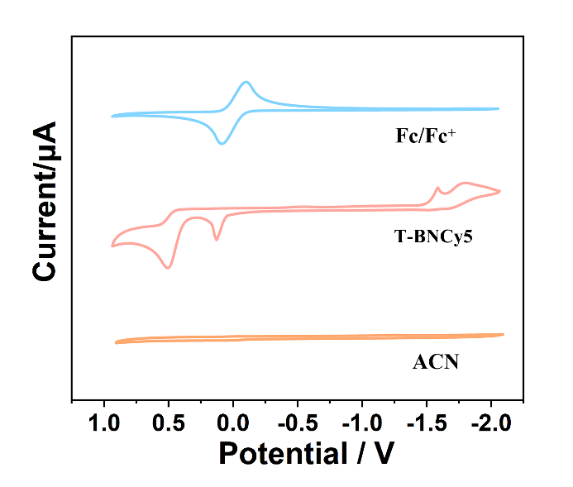


**Figure S4.** Cyclic voltammogram of **T-BNCy5**. Condition: in deaerated ACN containing 0.10 M Bu_4_N[PF_6_] as supporting electrolyte, Ag/AgNO_3_ as reference electrode. Ferrocene (Fc/Fc^+^) was used as the internal reference. Scan rates: 50 mVs^-1^.


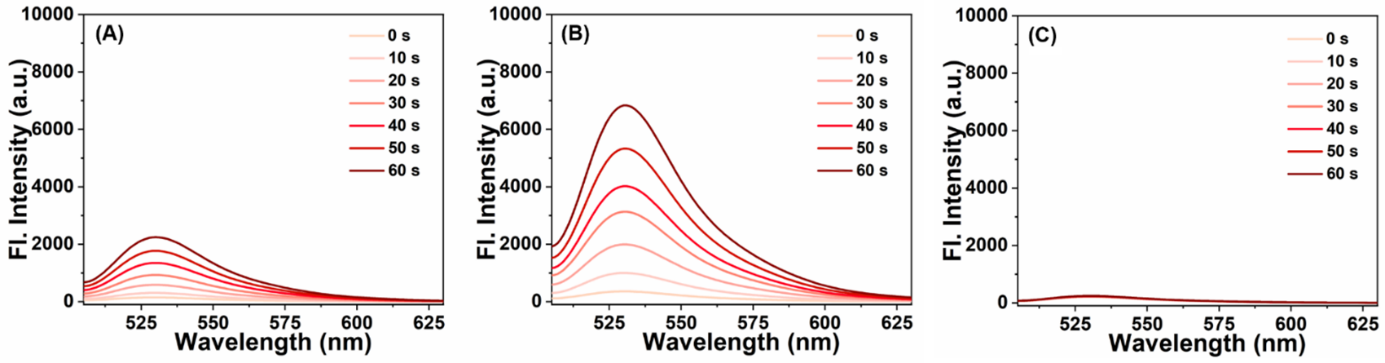


**Figure S5.** Fluorescence spectra changes of the water solutions of **ENCy5**/DHR123 (A), **BNCy5**/DHR123 (B), and DHR123 (C) under continuous irradiation of 660 nm LED light (20 mW cm^-2^). Concentrations for DHR123 and PS are 10 and 5 μM, respectively.


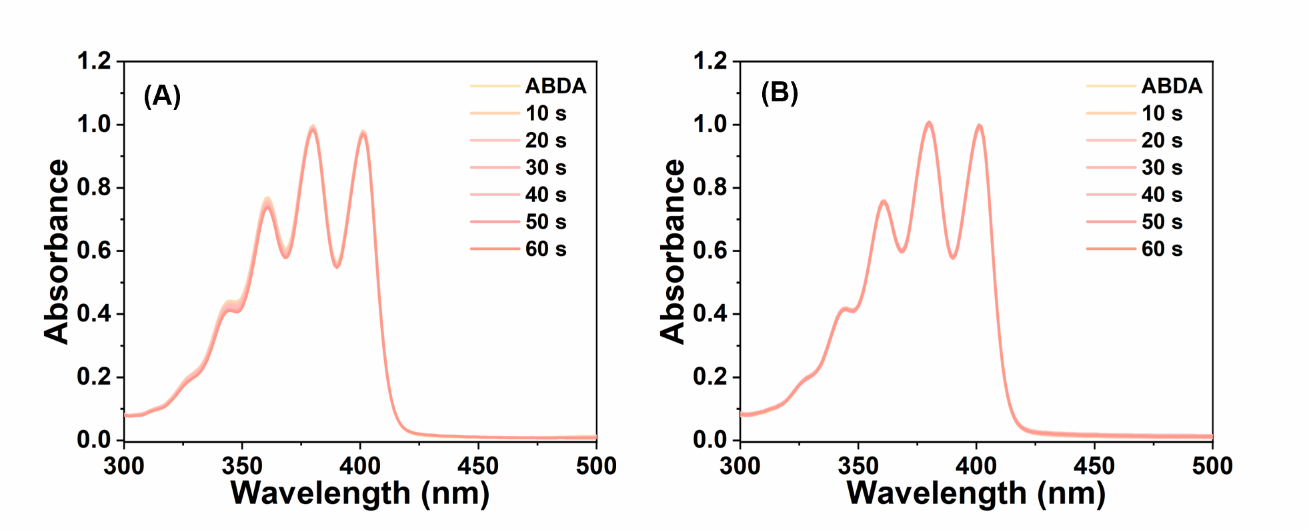


**Figure S6.** Absorption spectra changes of the PBS solutions of **ENCy5**/ABDA (A) and **BNCy5**/ABDA (B) under continuous irradiation of 660 nm LED light (20 mW cm^-2^).

**
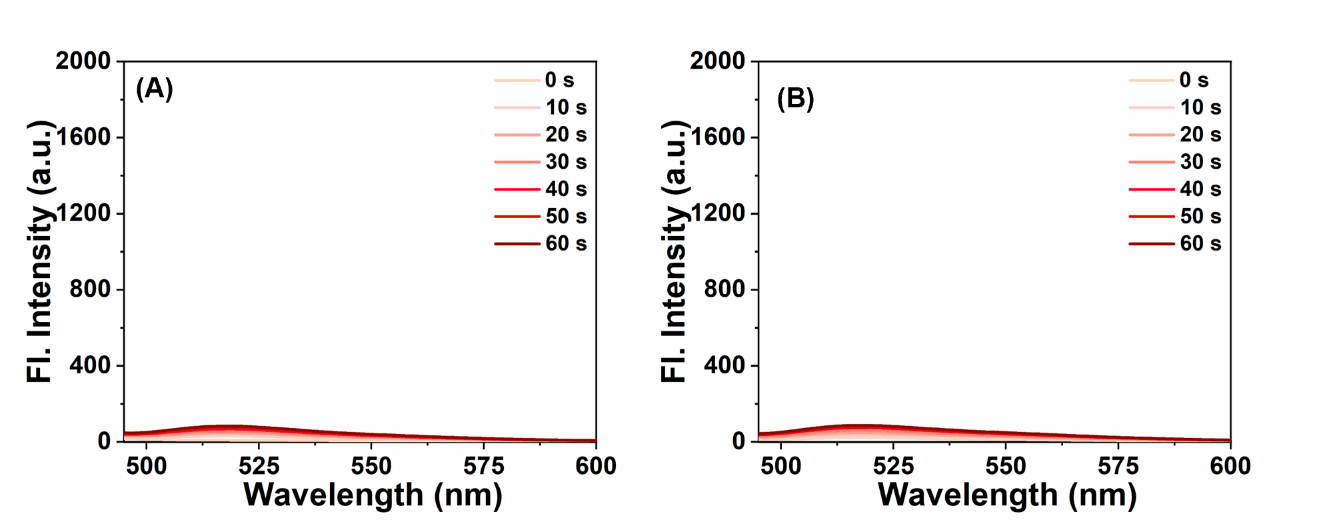
**

**Figure S7.** Fluorescence spectra changes of the water solutions of **ENCy5**/HPF (A) and (B) **BNCy5**/HPF under continuous irradiation of 660 nm LED light (20 mW cm^-2^). For HPF, excitation at 485 nm.


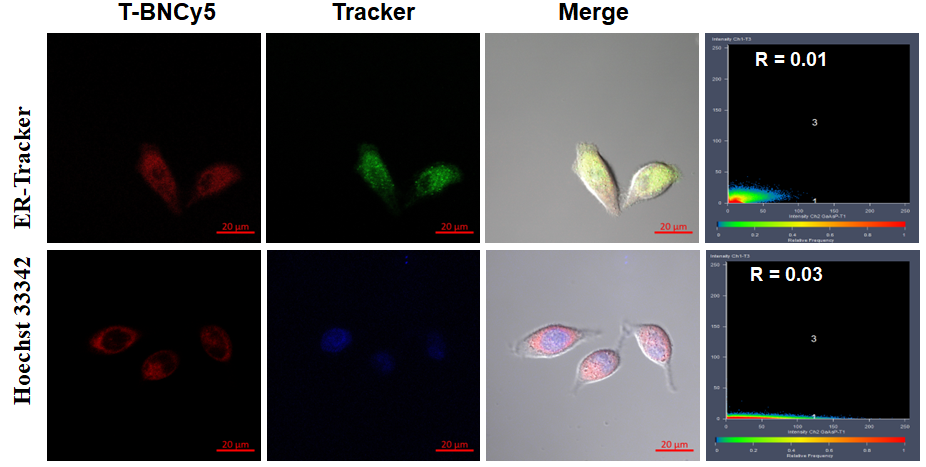


**Figure S8.** Confocal images of MCF-7 cells co-stained with **T-BNCy5**/ER-Tracker™ Green FM or **T-BNCy5**/Hoechst 33342. R refers to Pearson correlation coefficient.


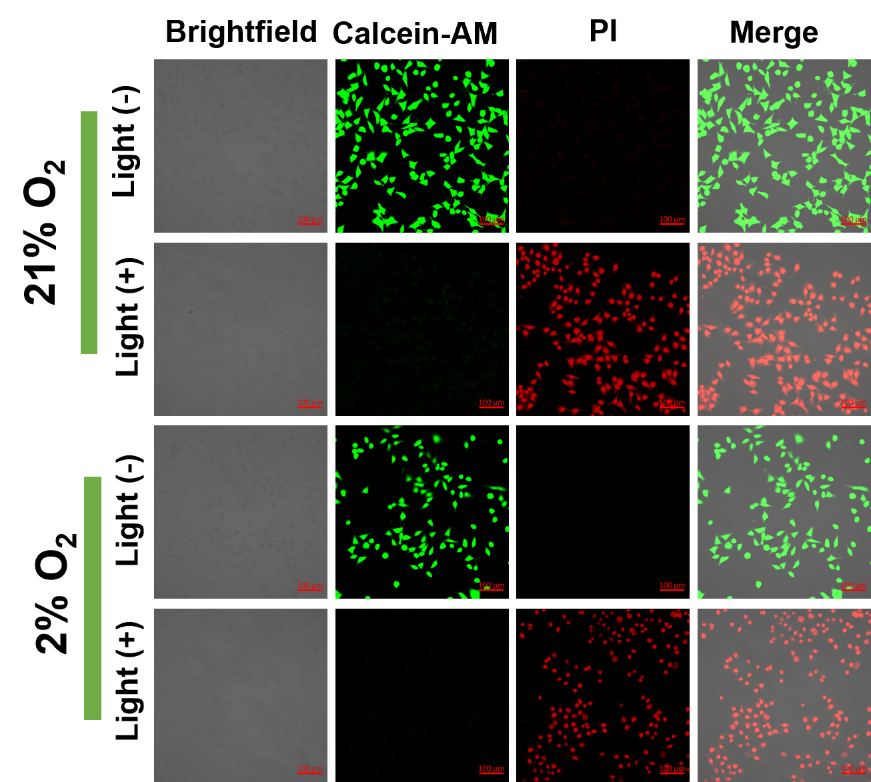


**Figure S9.** Confocal fluorescence images of MCF-7 cells co-stained with **T-BNCy5** (1.0 µM), Calcein-AM (5.0 µM), PI (1.0 µM) with and without continuous 660 nm LED light irradiation (20 mW cm^-2^, 20 min) under both normoxic (21% O_2_) and hypoxic (2% O_2_) conditions. For Calcein-AM, from 500 nm to 550 nm (λ_ex_ = 488 nm); for PI, from 570 nm to 620 nm (λ_ex_ = 561 nm).


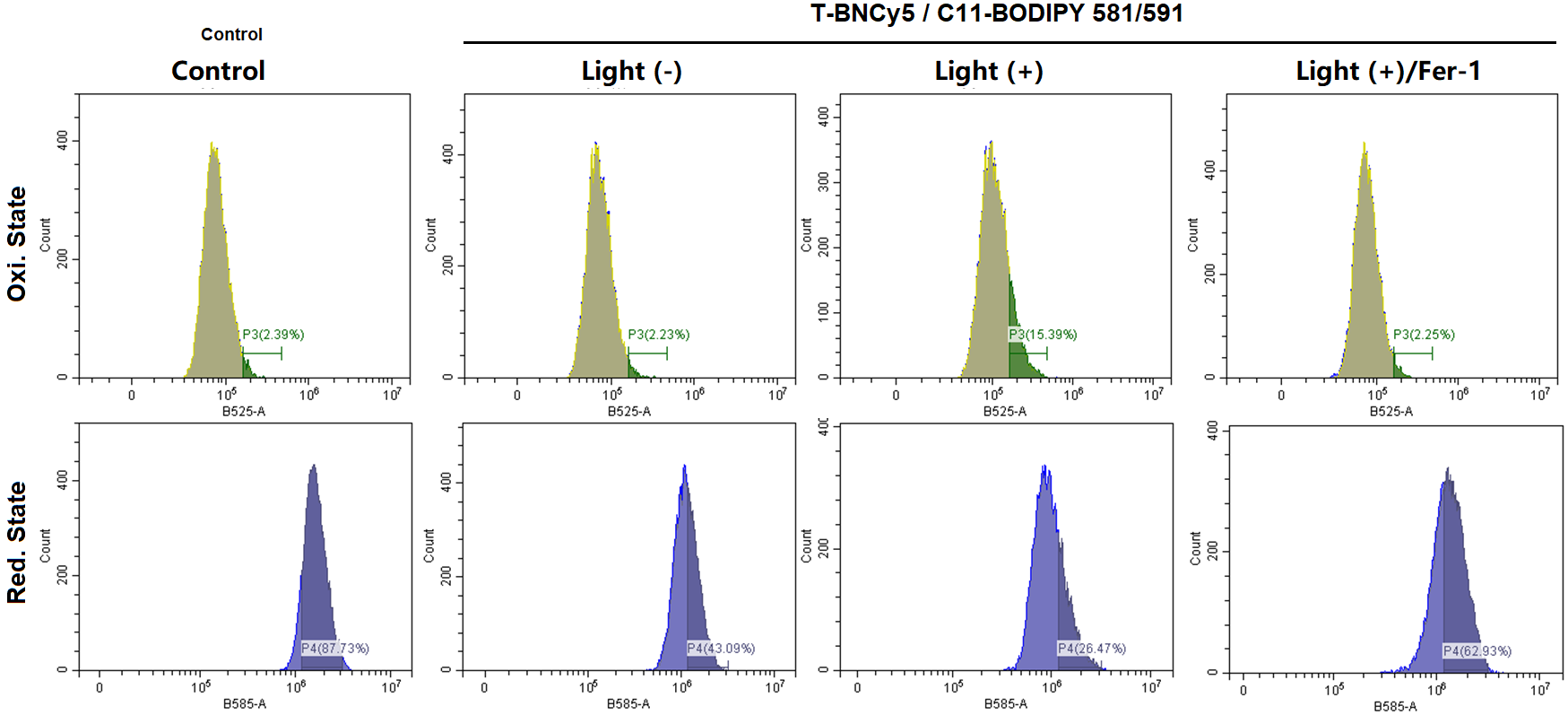


**Figure S10.** Flow cytometry analysis of MCF-7 cells co-stained with **T-BNCy5** (0.5 μM)/C11-BODIPY 581/591 in the absence and presence of Fer-1 and then treated with and without photoirradiation.


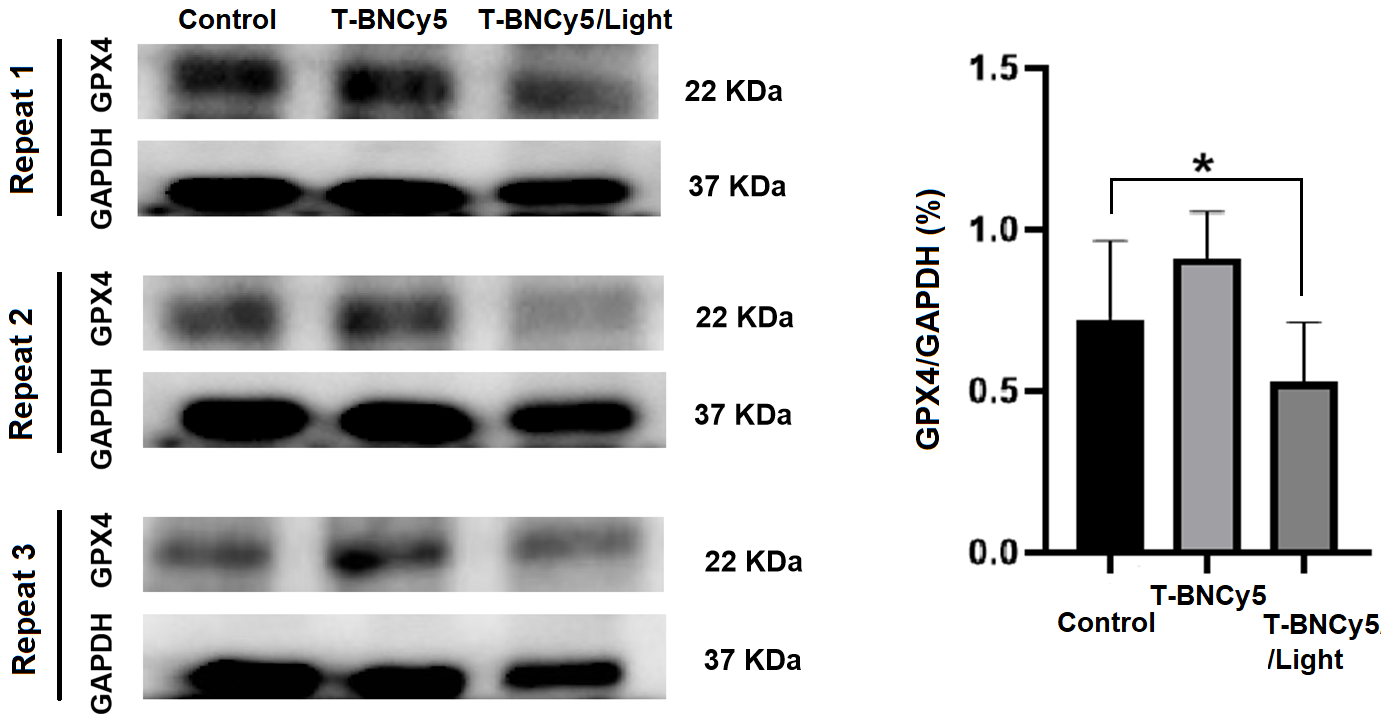


**Figure S11.** Western blot analysis of the expression of GPX4 in MCF-7 cells after the different treatments.


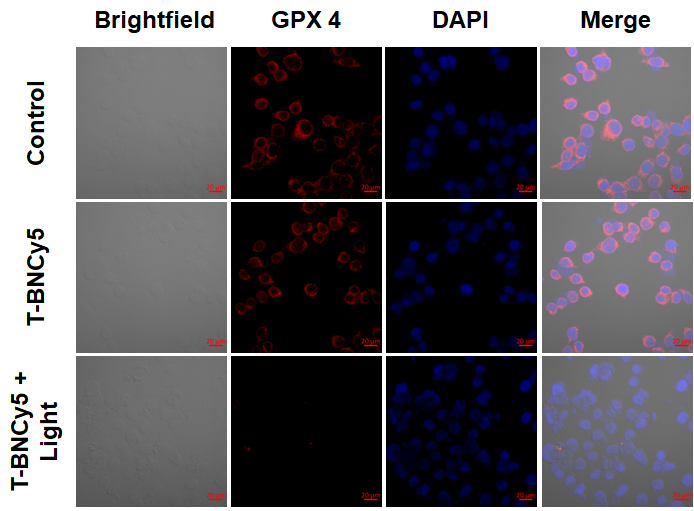


**Figure S12.** Immunofluorescence analysis of GPX 4 in MCF-7 cells after different treatments. (Scale bar = 20 μm).


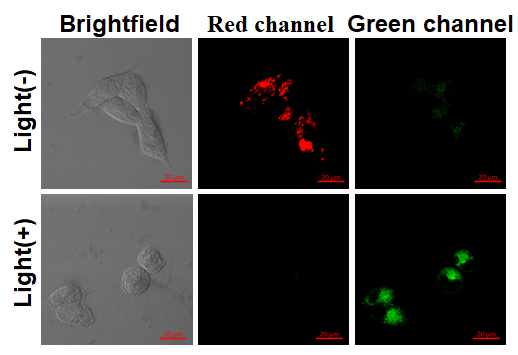


**Figure S13.** Confocal fluorescence images of MCF-7 cells co-stained with **T-BNCy5** (1.0 µM)/JC-1(10.0 µM) with and without continuous 660 nm LED light irradiation (20 mW cm^−2^, 20 min). Scale bar: 20 µm.


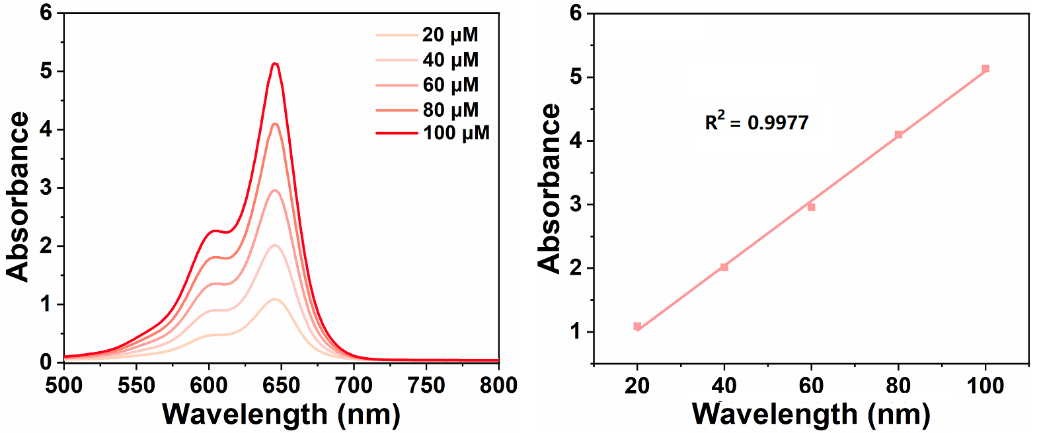


**Figure S14.** The concentration-dependent absorption spectra change of **T-BNCy5** (from 20 μM to 100 μM) in aqueous, which were obtained in a 1 mm path length quartz cuvette for ensuring absorbance within the range of UV-vis spectrophotometer. The result indicates that even in the concentration of 100 μM, no any aggregation of **T-BNCy5** could be observed. The aqueous solutions of **T-BNCy5** with different concentrations were prepared by adding its stock solution (2 mM in DMSO) into the 1 mL of deionized water.





**Figure S15.** Absorption spectra of **T-BNCy5** in water and urine.

**Figure S16.** HRMS spectrum of **T-BNCy5** in urine.


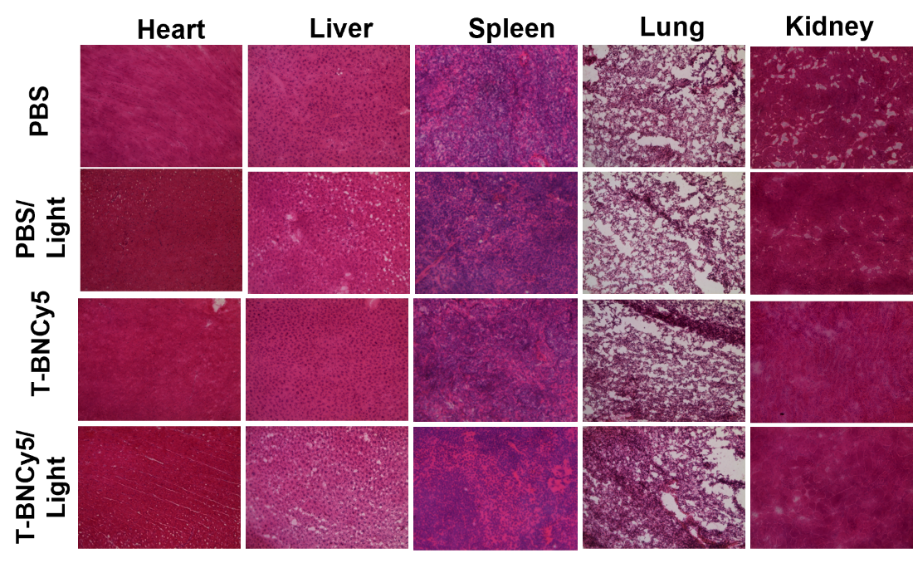


**Figure S17.** H&E staining of heart, liver, spleen, lung, and kidney of BALB/c mice after different treatments.


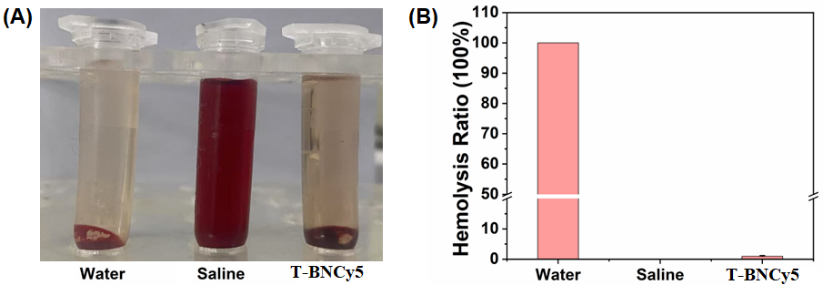


**Figure S18.** (A) Photographs of the hemolysis test in water (positive control), saline (negative control), and **T-BNCy5** (50μM in saline). (B) Hemolysis rates in water, saline, and **T-BNCy5**. Hemolysis assay was performed according to the reported procedures.^[11]^


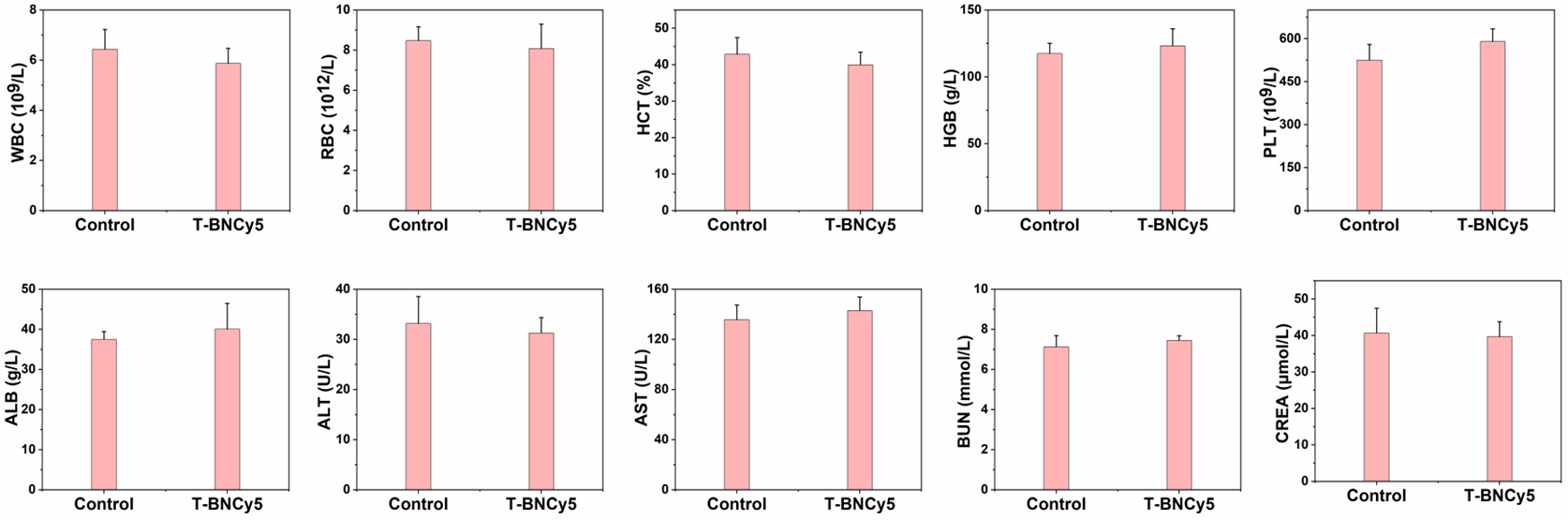


**Figure S19.** Hematological indexes [white blood cells (WBC), red blood cells (RBC), hematocrit (HCT), hemoglobin (HGB), and platelets (PLT)] and biochemical data [albumin (ALB), alanine aminotransferase (ALT), aspartate aminotransferase (AST), blood urea nitrogen (BUN), creatinine (CREA)] of mice injected with **T-BNCy5** and those not injected.


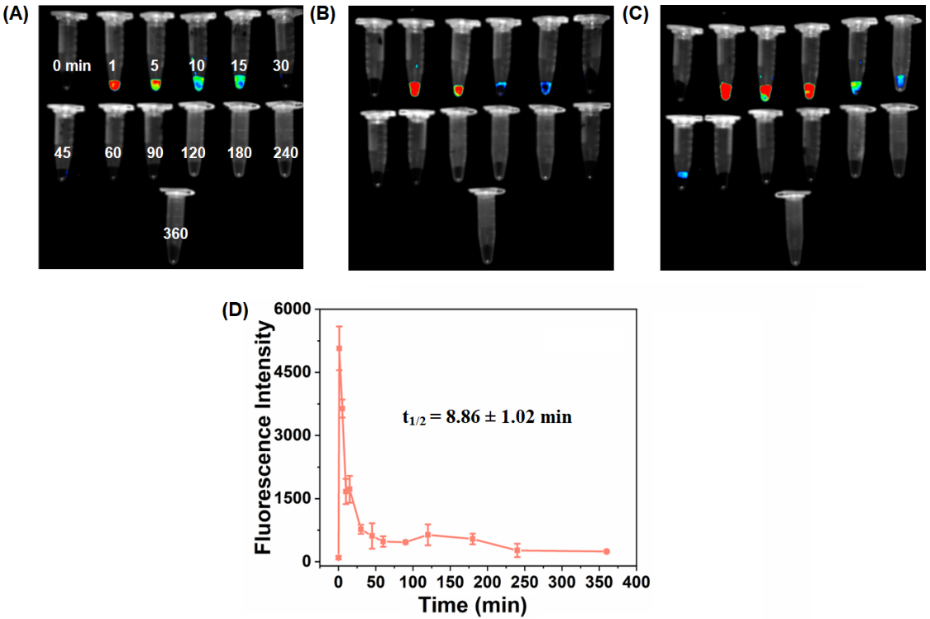


**Figure S20.** (A-C) Fluorescence images of the blood of three parallel 4T1 tumor-bearing mice, respectively, at the indicated time points after intravenous injection of **T-BNCy5**, and (D) the corresponding averaged fluorescence intensity changes.

**30. ^1^H NMR, ^13^C NMR and HRMS Charts**

**
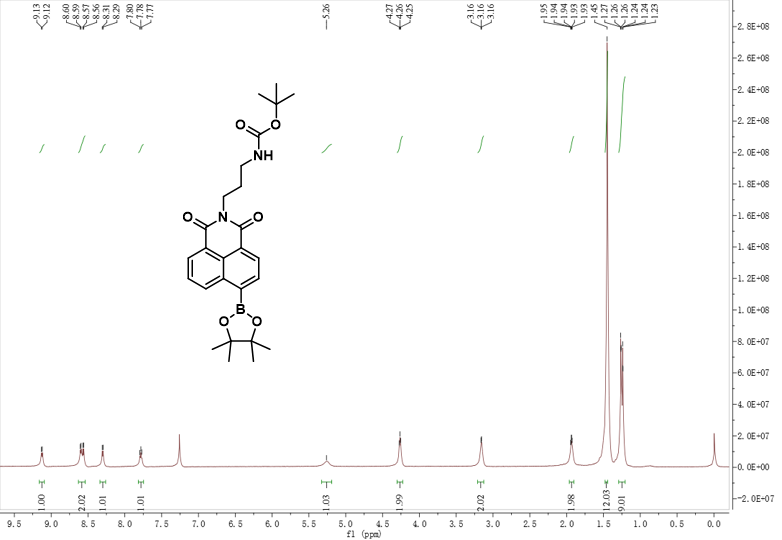
**

**Figure S21.** ^1^H NMR chart of Compound **4** (CDCl_3_, 600 MHz).


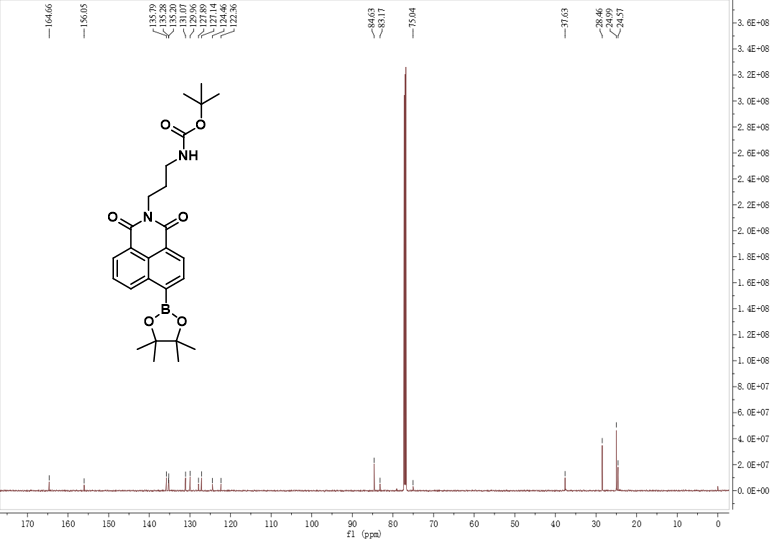


**Figure S22.** ^13^C NMR chart of Compound **4** (CDCl_3_, 151 MHz).


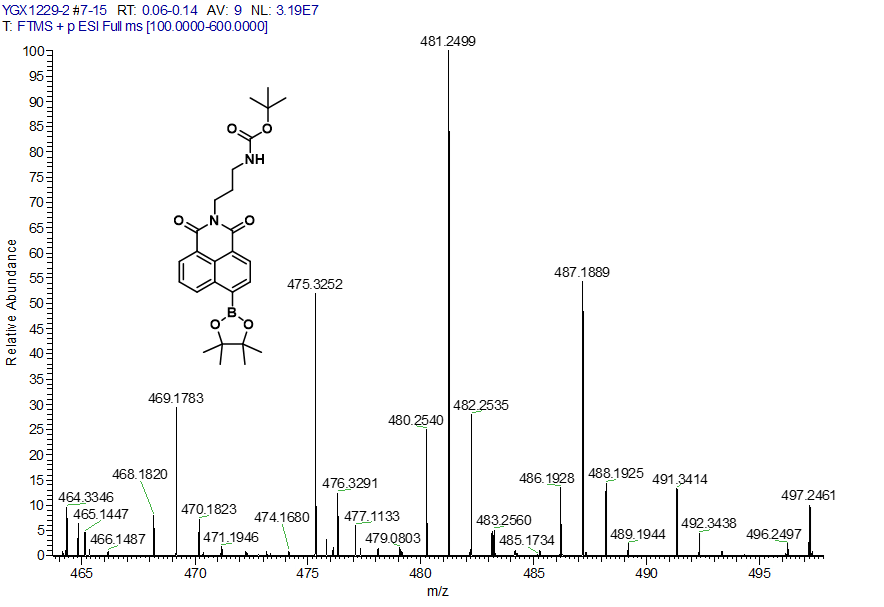


**Figure S23.** HRMS chart of Compound **4**.


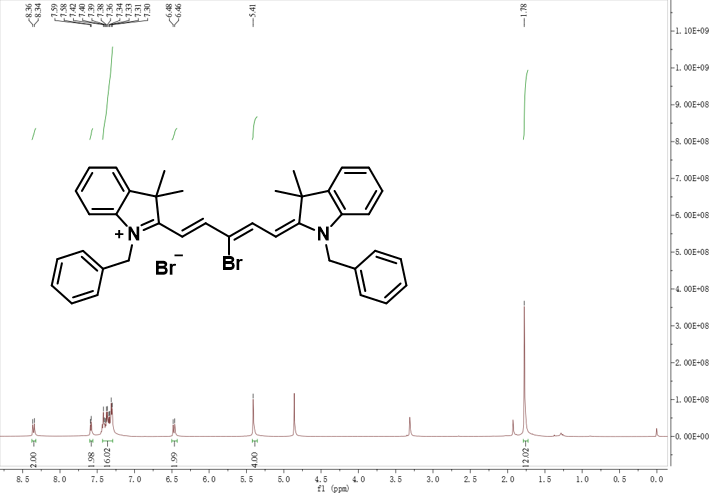


**Figure S24.** ^1^H NMR chart of **BCy5** (CD_3_OD, 600 MHz).


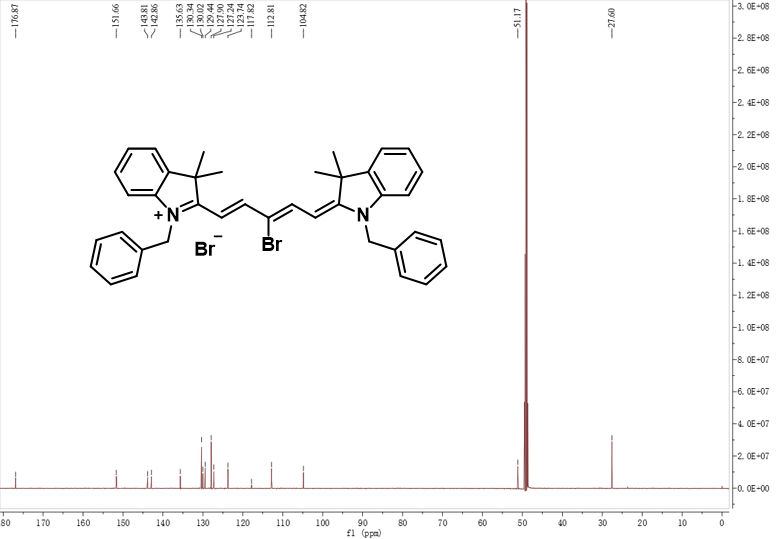


**Figure S25.** ^13^C NMR chart of **BCy5** (CD_3_OD, 151 MHz).

**
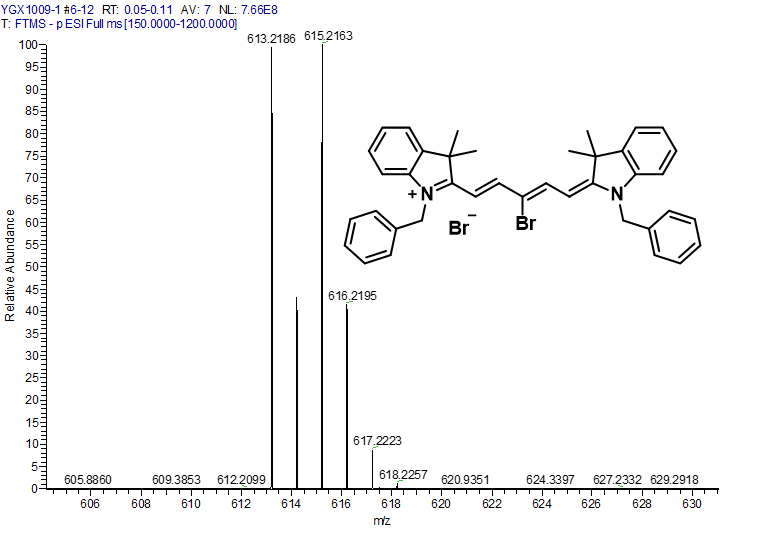
**

**Figure S26.** HRMS chart of **BCy5**.

**
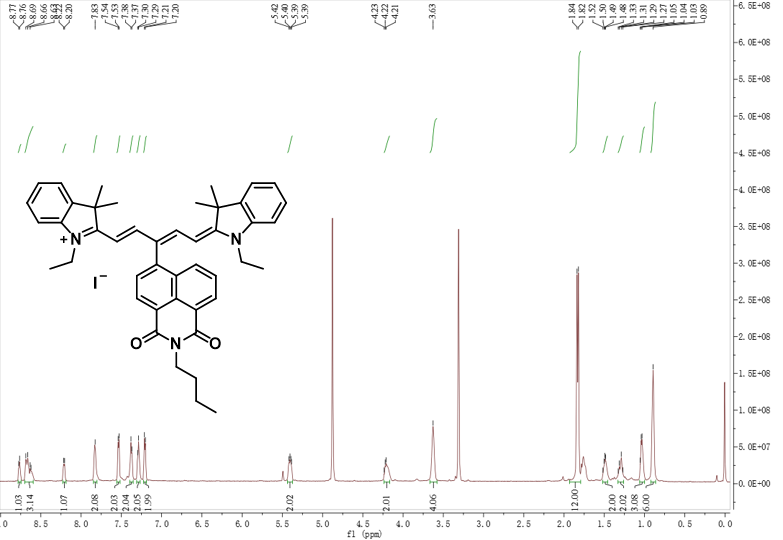
**

**Figure S27.** ^1^H NMR chart of **ENCy5** (CD_3_OD, 600 MHz).


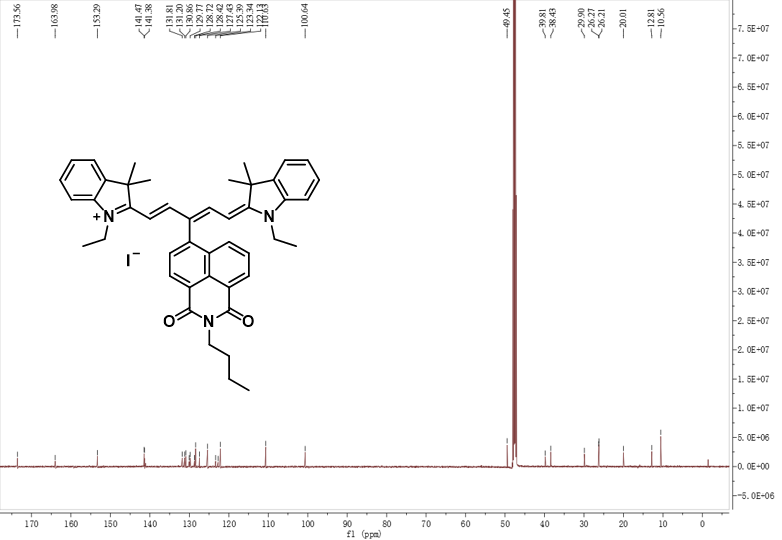


**Figure S28.** ^13^C NMR chart of **ENCy5** (CD_3_OD, 151 MHz).


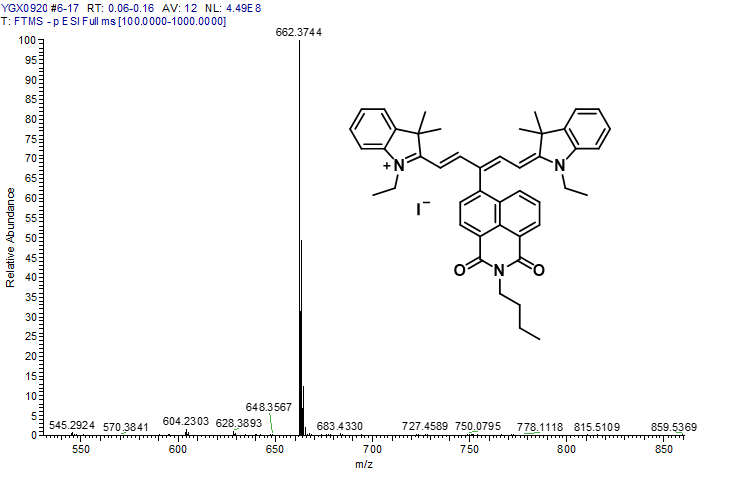


**Figure S29.** HRMS chart of **ENCy5**.


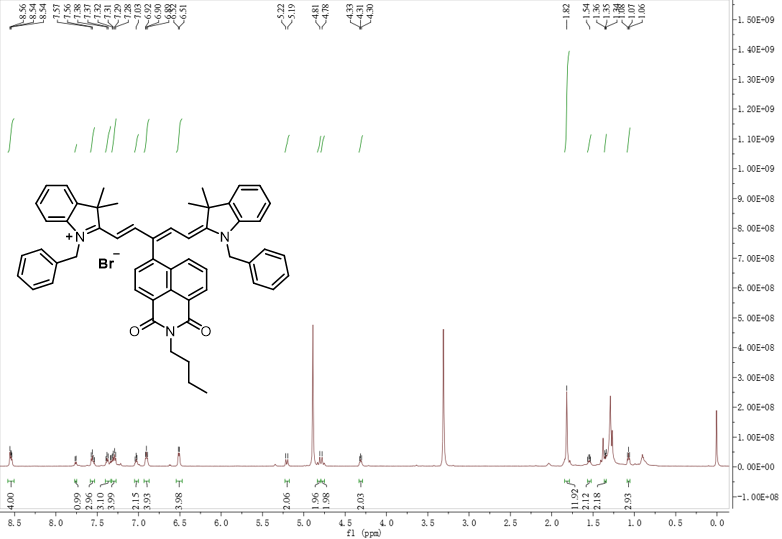


**Figure S30.** ^1^H NMR chart of **BNCy5** (CD_3_OD, 600 MHz).


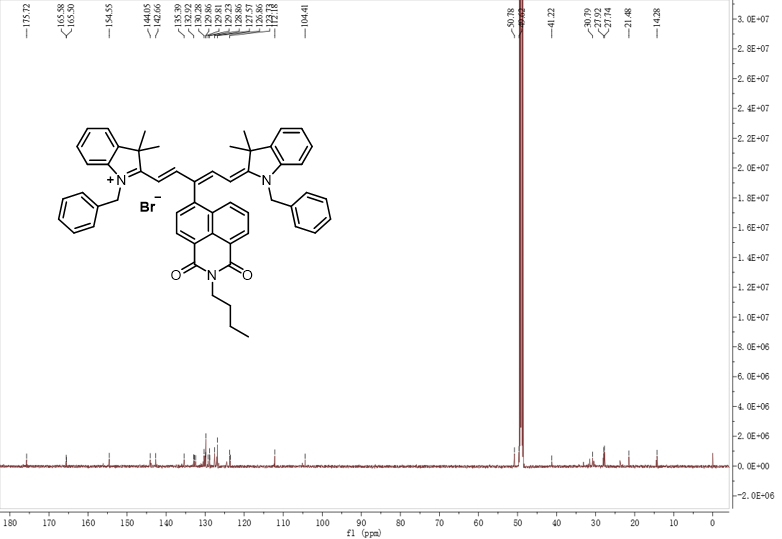


**Figure S31.** ^1^C NMR chart of **BNCy5** (CD_3_OD, 151 MHz).

**
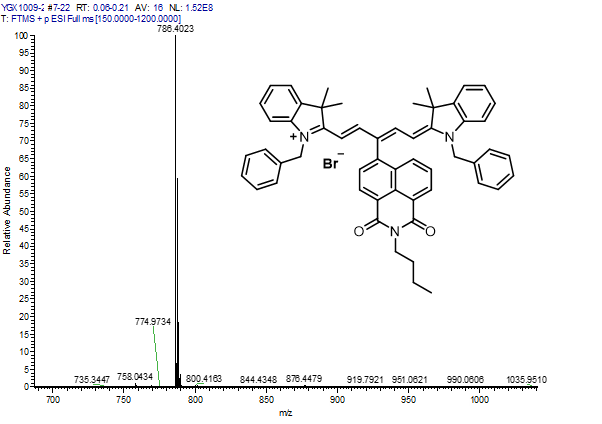
**

**Figure S32.** HRMS chart of **BNCy5**.


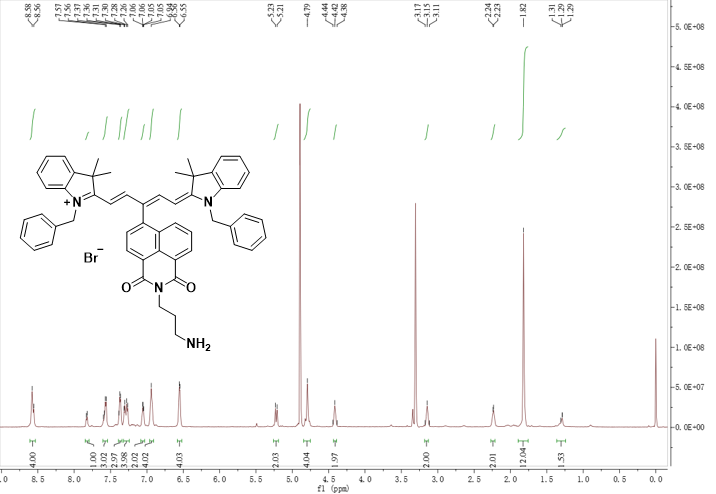


**Figure S33.** ^1^H NMR chart of **A-BNCy5** (CD_3_OD, 600 MHz).

**
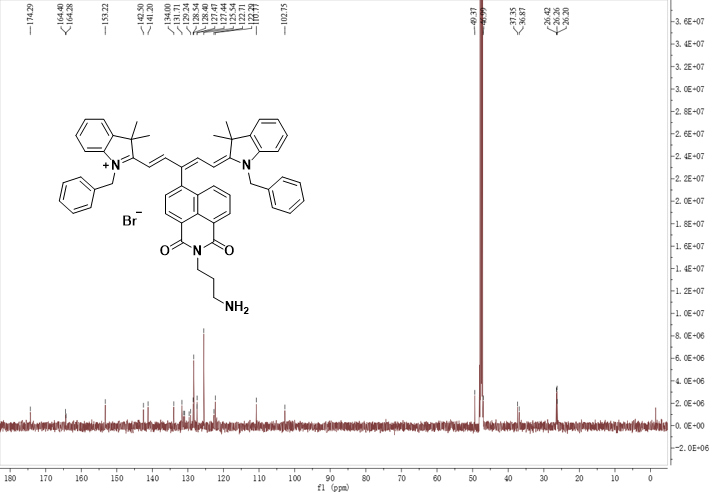
**

**Figure S34.** ^13^C NMR chart of **A-BNCy5** (CD_3_OD, 151 MHz).


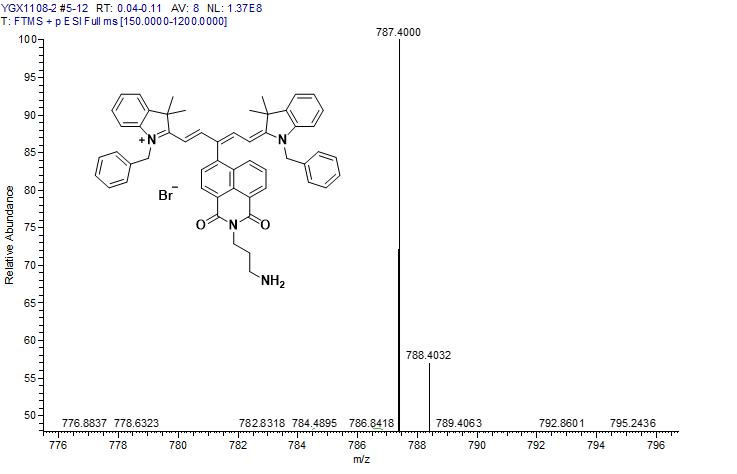


**Figure S35.** HRMS chart of **A-BNCy5**.


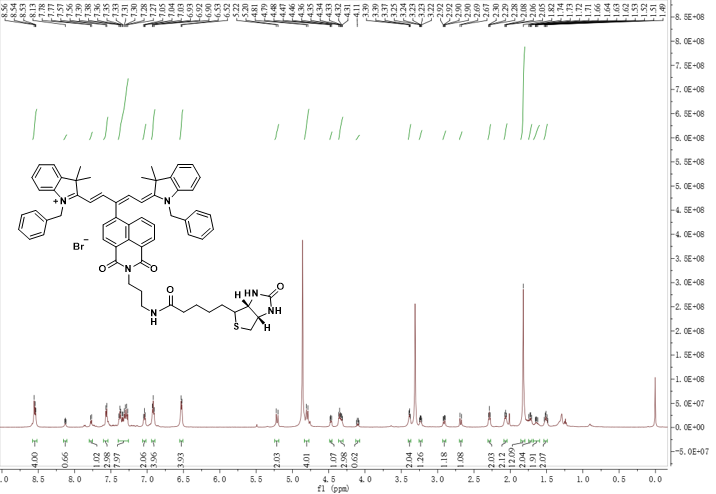


**Figure S36.** ^1^H NMR chart of **T-BNCy5** (CD_3_OD, 600 MHz).


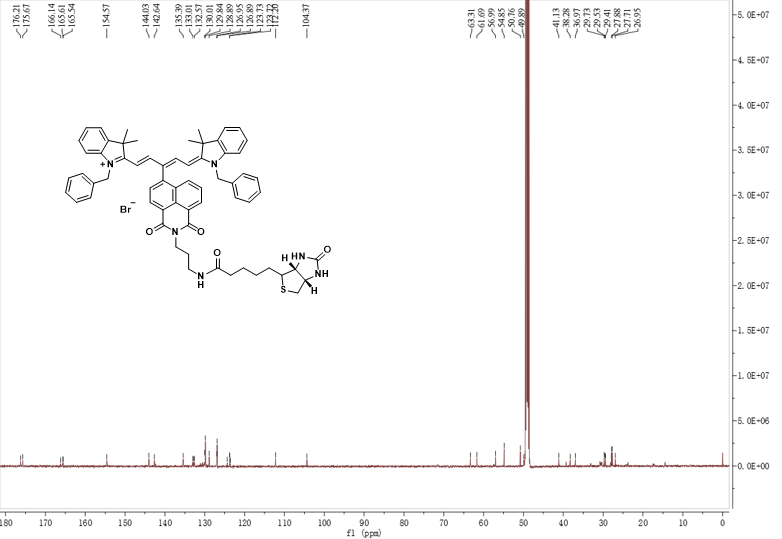


**Figure S37.** ^13^C NMR chart of **T-BNCy5** (CD_3_OD, 151 MHz).


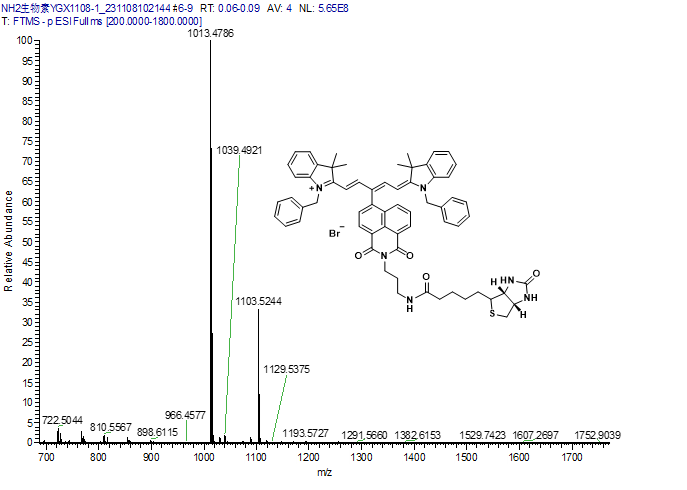


**Figure S38.** HRMS chart of **T-BNCy5**.

**References**

[1] J. Zhuang, B. Wang, H. Chen, K. Zhang, N. Li, N. Zhao, B. Z. Tang, *ACS Nano* **2023**, *17*, 9110-9125.

[2] K.-X. Teng, L.-Y. Niu, Q.-Z. Yang, *J. Am. Chem. Soc.* **2023**, *145*, 4081-4087.

[3] K.-X. Teng, L.-Y. Niu, N. Xie, Q.-Z. Yang, *Nat. Commun.* **2022**, *13*, 6179.

[4] K. X. Teng, W. K. Chen, L. Y. Niu, W. H. Fang, G. Cui, Q. Z. Yang, *Angew. Chem. Int. Ed.* **2021**, *60*, 19912-19920.

[5] X. Chen, A. A. Sukhanov, Y. Yan, D. Bese, C. Bese, J. Zhao, V. K. Voronkova, A. Barbon, H. G. Yaglioglu, *Angew. Chem. Int. Ed.* **2022**, *61*, e202203758.

[6] W. Tian, A. A. Sukhanov, L. Bussotti, J. Pang, J. Zhao, V. K. Voronkova, M. Di Donato, M.-D. Li, *J. Phys. Chem. B* **2022**, *126*, 4364-4378.

[7] J. Miao, G. Yao, Y. Huo, B. Wang, W. Zhao, W. Guo, *ACS Appl. Mater. Interfaces* **2024**, *16*, 40428-40443.

[8] K. X. Teng, D. Zhang, B. K. Liu, Z. F. Liu, L. Y. Niu, Q. Z. Yang, *Angew. Chem. Int. Ed.* **2024**, *63*, e202318783.

[9] M. Li, J. Xia, R. Tian, J. Wang, J. Fan, J. Du, S. Long, X. Song, J. W. Foley, X. Peng, *J. Am. Chem. Soc.* **2018**, *140*, 14851-14859.

[10] Q. Ren, H. Wang, D. Li, A. Dao, J. Luo, D. Wang, P. Zhang, H. Huang, *Adv. Healthc. Mater* **2024**, *13*, 2304067.

[11] Z. Xu, X. Li, Z. Yang, Z. Zhang, Y. Zhang, M.g Fan, Y. Zeng, M. Kang, Y. Shen, D. Wang, G. Xu, B. Z. Tang, *Adv. Mater.* **2024**, 2413164.
